# Multi‐Dimensional Multiplexed Metasurface for Multifunctional Near‐Field Modulation by Physics‐Driven Intelligent Design

**DOI:** 10.1002/advs.202503899

**Published:** 2025-04-29

**Authors:** Jian Lin Su, Zi Xuan Cai, Yiqian Mao, Long Chen, Xin Yi Yu, Zhi Cai Yu, Qian Ma, Si Qi Huang, Jianan Zhang, Jian Wei You, Tie Jun Cui

**Affiliations:** ^1^ State Key Laboratory of Millimeter Wave Southeast University Nanjing 210096 China; ^2^ Institute of Electromagnetic Space Southeast University Nanjing 210096 China; ^3^ Suzhou Laboratory Suzhou Jiangsu 215000 China

**Keywords:** coupled mode theory, multiple degrees of freedom, multiplexed metasurfaces, near‐field manipulation, physics‐driven intelligent design

## Abstract

Metasurface is a revolutionary platform to achieve desired properties by artificially engineering meta‐atom's arrangements. However, the explosively expanding design space of advanced metasurfaces with multiple degrees of freedom (MDOF) has made the traditional human‐guided design methods increasingly ineffective, limiting the development of the metasurfaces. Intelligent design methods have been presented to tackle these challenges by introducing innovative computational models, but they are predominantly data‐driven and faced the issues of data scarcity, poor physical interpretability, and weak generalization capability. Here, a physics‐driven intelligent design (PDID) paradigm is proposed and demonstrates its application to design MDOF multiplexed metasurfaces. The PDID method integrates the physical prior knowledge into a deep neural network, thereby enhancing its physical interpretability and reducing its reliance on extensive databases. Compared to the traditional intelligent designs, this can reduce both design time and database size by two orders of magnitude. Through experimental validation of MDOF multiplexed metasurfaces, the versatility and computational efficiency of PDID are showed. This method not only presents a novel intelligent design tool but also exemplifies the integration of physical knowledge with machine learning to address the challenges. Its interdisciplinary insights offer significant potentials for innovative applications across the materials science, computational science, and information technology.

## Graphical abstract

The expansive design space of advanced metasurfaces, particularly those with multiple degrees of freedom (MDOF), renders traditional design methods less effective. A physics‐driven intelligent design is proposed for MDOF metasurfaces, which integrates physical knowledge into neural networks, enhancing interpretability and computational efficiency. This method significantly reduces both design time and database size.

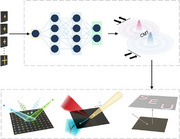

## 1 Introduction

Over the past few decades, metasurface technology has sparked a revolutionary wave in the field of electromagnetic (EM) manipulation.^[^ ^1^, ^2^, ^3^, ^4^, ^5^, ^6^ ^]^ Through the ingenious design of individual meta‐atoms and their arrangement, the metasurfaces have achieved on‐demand control over their EM responses, thereby giving rise to a series of EM functionalities, including EM lenses, holography, and beam forming.^[^ ^7^, ^8^, ^9^, ^10^, ^11^, ^12^, ^13^, ^14^, ^15^, ^16^, ^17^ ^]^ Ref. [17] employs a topology optimization method to design an optical metasurface for optical routing and rigorously analyzes its performance using Rigorous Coupled Wave Analysis (RCWA) and overcomes the efficiency limitations of conventional color filter architectures. With the increasing demands for the precision and complexity of EM manipulations, the sizes and multiple degrees of freedom (MDOF) of metasurfaces are constantly being explored. To this end, a multitude of innovative design solutions have been proposed, aimed to design multifunctional metasurfaces with high design precision and minimal computational cost.^[^ ^18^, ^19^, ^20^, ^21^ ^]^ Among them, an inverse design strategy based on numerical calculations has emerged for metasurfaces. This strategy rigorously calculates the EM response of metasurfaces and iterates continuously through optimization algorithms until the predetermined design objectives are met. Numerical models and methods play important roles in the design of metasurfaces due to their high interpretability and versatility, but they are often computational expensive.

The emergence of artificial intelligence (AI) has triggered a paradigm shift in the metasurface design, with the application of deep neural networks becoming a prevailing methodology.^[^ ^22^, ^33^ ^]^ Inverse design techniques aided by neural networks, such as multilayer perceptron (MLP),^[^ ^22^, ^29^ ^]^ generative adversarial network (GAN),^[^ ^23^, ^30^ ^]^ and neural adjoint (NA),^[^ ^31^ ^]^ have achieved remarkable success in metasurface designs, demonstrating immense potentials. Ref. [33] introduces the REDCNN method, which enables the direct prediction of complex amplitude holographic metasurface layouts from target holographic fields. The above studies employ data‐driven neural networks, commonly referred to as the “black box”. While these networks have been shown to significantly reduce the design time, the lack of prior knowledge resulted in the loss of physical interpretability. As the neural networks become increasingly complicated, the establishment of comprehensive datasets becomes more challenging. To address these challenges, it is crucial to integrate the prior physical knowledge into the prediction process of neural networks.^[^ ^34^, ^35^, ^36^, ^37^ ^]^ Incorporating physical equations into the design process, thereby partially revealing the “black box”, is expected to significantly alleviate the training burden of neural networks. In this way, it is possible to improve the accuracy and interpretability of designs without sacrificing the design efficiency, thereby promoting further advancements in designing metasurfaces.

In this study, we present a physics‐driven intelligent design (PDID) method for the inverse design of the MDOF multiplexed metasurfaces. By integrating the coupled mode theory (CMT) equations with the neural networks, our approach is capable of calculating the near‐field characteristics of arbitrary large‐scale MDOF multiplexed metasurface. In contrast to the methods presented in refs. [17, 33] the PDID framework uniquely integrates physical prior knowledge into neural networks, effectively reducing data requirements while preserving high design efficiency. By embedding fundamental physical principles directly into the learning process, PDID not only enhances predictive accuracy but also accomplishes more complicated tasks to achieve more sophisticated EM wave manipulation. Based on this approach, we theoretically design and experimentally illustrate several large‐scale MDOF multiplexed metasurfaces. The integration of physical laws into neural networks has been validated to address the computationally expensive challenges of the traditional numerical methods and to alleviate the dataset intensive burden of the data‐driven intelligent design methods.

## 2 Results

### 2.1 The System

**Figure 1:**
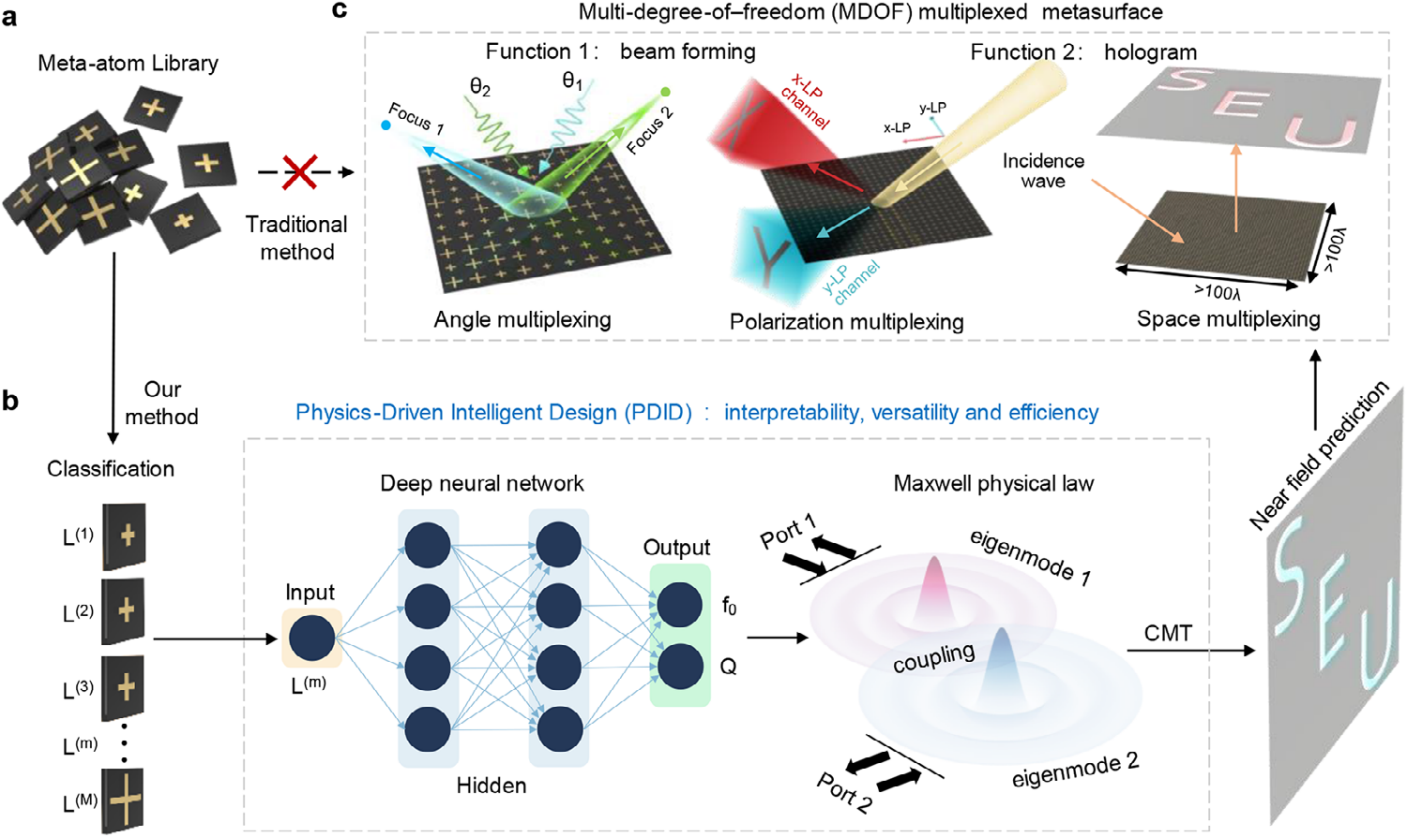
Scheme of the MDOF multiplexed metasurface and its PDID method. a) Illustration of a meta‐atom library. b) The PDID method to integrate the physical law, namely CMT, into the deep neural network. c) The MDOF multiplexed metasurface designed by the PDID method to implement beam forming and holography without relying on computationally expensive numerical simulations.

The MDOF multiplexed metasurface comprises an array of cross‐shaped meta‐atoms, as shown in **Figure** **1**a. To enable efficient and accurate prediction of near‐field characteristics of large‐scale MDOF multiplexed metasurfaces, we introduce a physical law, namely the coupled mode theory for Maxwell's equations, into the neural network to develop a physics‐driven intelligent design, as illustrated in Figure 1b. In the PDID method, the neural‐network layer is used to predict the EM responses of each meta‐atom, while the mutual‐coupling effect of meta‐atoms and the near‐field distribution are evaluated by the CMT‐based physical law layer. More specifically, the input and output of the pre‐trained neural network are geometric parameters and electromagnetic responses (e.g., resonant frequency *f*_0_ and quality factor *Q*) of each meta‐atom, respectively. The predicted electromagnetic responses are subsequently incorporated into the coupled mode theory to accurately and efficiently evaluate the near‐field distributions of the MDOF multiplexed metasurfaces, by taking into account the mutual coupling effects of the arranged meta‐atoms. See Supporting Information for details.

Compared to the previous data‐driven intelligent design, this approach incorporates a CMT‐based physical law layer into the neural network to enhance the interpretability of the design framework. Moreover, the traditional data‐driven intelligent designs typically require large amounts of metasurface datasets for training the neural networks, and a new database and a new training process are required to design these metasurfaces, which are not included in the metasurface datasets, leading to an unacceptable design complexity. This challenge can be well addressed by the proposed PDID method, as its training process only requires a small amounts of meta‐atom datasets rather than large amounts of metasurface datasets. Metasurfaces of arbitrary shapes and arrangements can be constructed from the meta‐atom database, eliminating the need to rebuild the database for different sizes and functionalities. This method not only enhances the versatility of design but also dramatically reduces its complexity, and such superiority can be used to significantly facilitate the development of MDOF multiplexed metasurfaces. The rich degrees of freedom (DOFs) of EM waves are valuable resources that have long attracted significant attention. However, the manipulation of multiple DOFs on a single metasurface remains one of the major challenges in this field. The primary reason is that controlling multiple DOFs involves to optimize multiple objectives, while the meta‐atoms within metasurfaces exhibit strong nonlinear coupling. These challenges significantly increase the complexity to design the metasurfaces with MDOF multiplexing. In the following sections, we will detail the theoretical and experimental results to demonstrate the great effectiveness of the PDID method in addressing these challenges.

### 2.2 Intelligent Optimization

**Figure 2:**
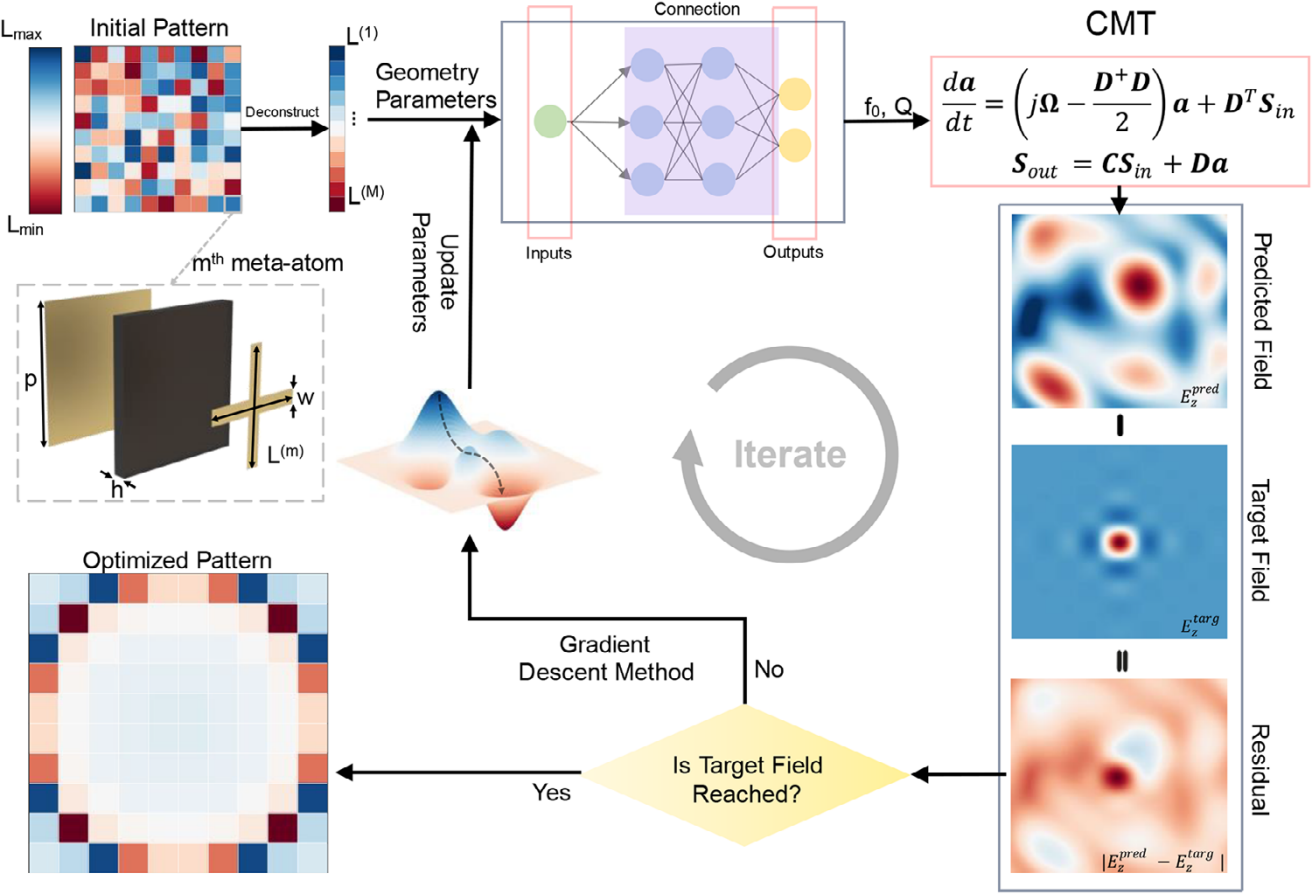
The PDID‐based optimization of metasurfaces for near‐field manipulations. The optimization process begins with the generation of a random pattern of meta‐atoms. The geometric parameters of each meta‐atom are input to a pre‐trained neural network to predict its resonant frequency *f*_0_ and quality factor *Q*. These EM parameters are then incorporated into the Coupled Mode Theory (CMT) equations to predict the near‐field distribution. The predicted near field is compared to the target field, and the geometric parameters and arrangement of meta‐atoms are optimized via gradient descent until the desired performance is achieved.

The PDID‐based optimization of metasurfaces for near‐field manipulation is illustrated in **Figure** **2**, where each meta‐atom consists of three layers. The top layer features a cross‐shaped metallic patch with arm length *L* and width *w*, the middle layer is a dielectric substrate of thickness *h* and the period of the meta‐atom is *p*. The design process begins with the generation of a random metasurface, which is subsequently deconstructed into individual meta‐atoms. The geometric parameters of each meta‐atom are input to a pre‐trained neural network to predict its EM parameters *f*_0_ and *Q*. These EM parameters are then integrated into the CMT equations to predict the near‐field distribution of the input metasurface. Then, the predicted near‐field distribution is compared with the target field. If the target is not reached, the geometric parameters and arrangement of the meta‐atoms are iteratively optimized using gradient descent. The PDID method utilizes the adjoint method for gradient computation, which efficiently reduces the complexity associated with calculating the derivatives of the loss function (Note S11, Supporting Information, for details). By introducing adjoint variables, the method avoids direct differentiation with respect to all parameters, thereby significantly enhancing computational efficiency while maintaining accuracy. This iterative process continues until the target is reached.

### 2.3 Intelligent Design

**Figure 3:**
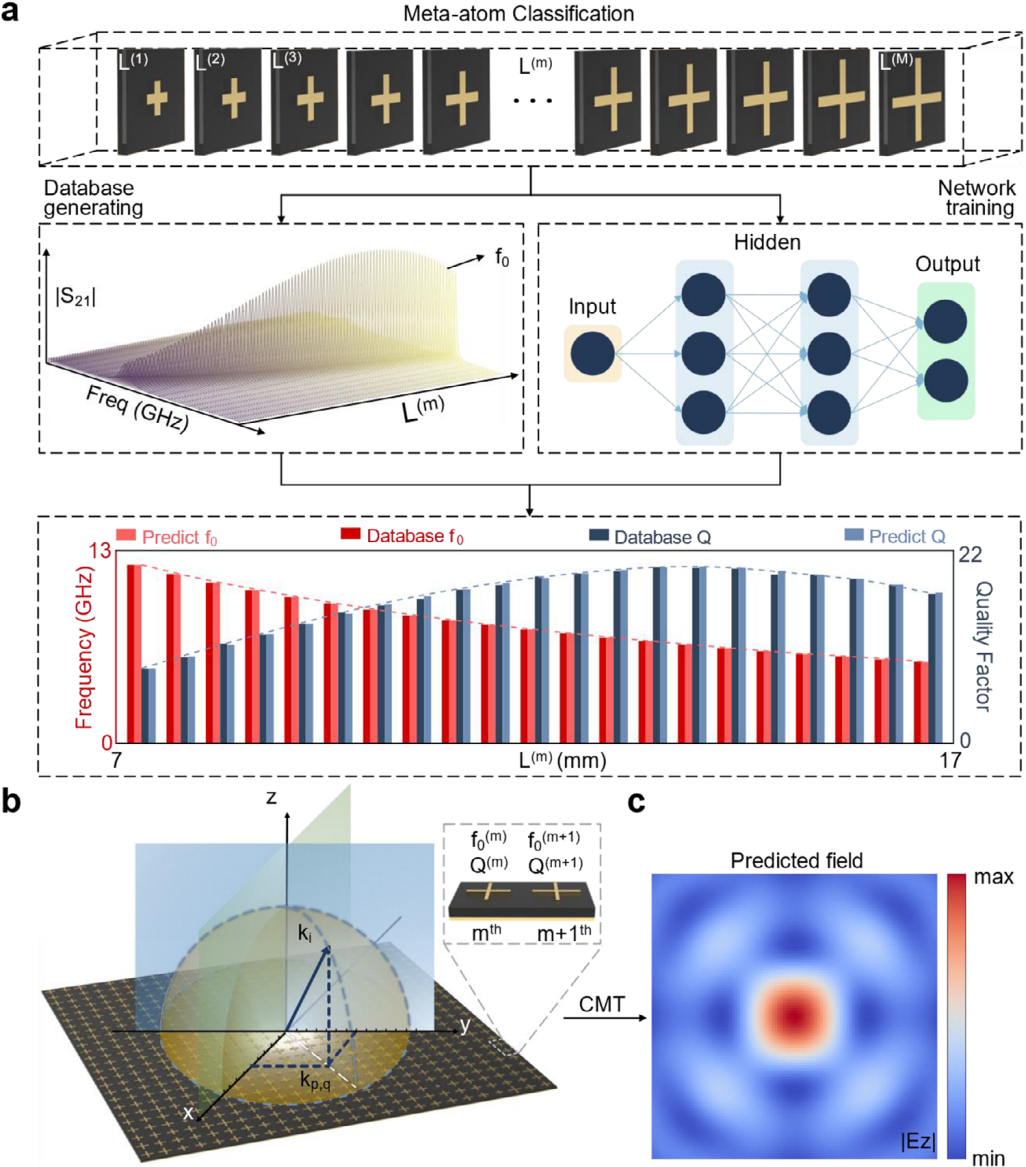
Near‐field prediction based on PDID. a) Demonstration of the neural network training process using a meta‐atom library to predict the EM parameters (resonant frequency f_0_ and quality factor Q) of each meta‐atom. b) By considering the mutual‐coupling effect of meta‐atoms, a physical law algorithm based on CMT is used to decompose the incident and scattered waves into orthogonal channels and compute the near‐field distributions. c) Near‐field prediction of an optimized metasurface designed by PDID is shown to achieve the desired light focusing function, demonstrating the high accuracy and efficiency of PDID.

Prior to training the network, a database termed as the meta‐atom library was constructed, as shown in **Figure** **3**a. This library was established through the EM numerical simulations, which were used to obtain the S‐parameters of meta‐atoms with varying geometric dimensions *L*  = {*L* ^(1)^,*L* ^(2)^,⋅⋅⋅, *L* ^(*n*)^,⋅⋅⋅, *L* ^(*M*)^}. For each meta‐atom, the resonant frequency *f*_0_ is directly extracted from the simulated S‐parameters and the quality factor *Q* was calculated by $Q=\frac{f_{0}}{\Delta f}$, where Δ*f* is half‐power bandwidth. The obtained resonant frequency *f*_0_ and quality factor *Q* of the n^th^ meta‐atom are correlated with the geometric parameter *L* ^(*n*)^, thereby enabling the construction of the meta‐atom library. Subsequently, this meta‐atom library serves as the database for training a neural network. The comparison between the simulated and predicted results is given in Figure 3a. The close agreement between the predicted and simulated results demonstrates the high accuracy of the network's predictions.

In the proposed PDID method, the physics‐law layer is constructed using the coupled mode theory. As depicted in the top left corner of Figure 3b, the incident and scattered waves are decomposed into *n* orthogonal channels, denoted by *S_in_* and *S_out_*, respectively. Each vector has a length of *n*. This decomposition allows the incident wave, regardless of its angle of incidence, to be separated into multiple orthogonal channels for computational analysis. This process enables the efficient determination of the corresponding scattered wave. The scattered near‐field intensity can then be calculated by *F* (*f*, *r*′) = |*W*(*r*′)*S_out_* (*f*)|^2^. Here, *f* represents the incident frequency, *r*′ = (*x*′, *y*′, *z*′) denotes the position at which the field intensity is calculated, and $W\;\left(r^{\prime }\right)=\left(e^{-jk_{1}\cdot r^{\prime }},e^{-jk_{2}\cdot r^{\prime }},\text{…},e^{-jk_{n}\cdot r^{\prime }}\right)$ represents the propagation phases of the plane waves in each channel. To address the issue of channel decomposition, this work introduces the Periodic Boundary Condition (PBC) as an effective approach, enabling precise control over the number of channels while maintaining computational accuracy. By incorporating a virtual period *a* into a non‐periodic array, we ensure a structured representation of wave propagation. As rigorously demonstrated in ref. [38], for a periodic structure, an incident plane wave can be decomposed into several discrete plane wave channels, which are uniformly distributed in *k*‐space with a spacing of $\Delta k=\lfloor \frac{2\pi }{a}\rfloor$. Considering the in‐plane component *k*_*p*,*q*, *i*_ of the wave vector *k_i_* for the *i*‐th channel in the *xoy*‐plane, it satisfies $k_{p,q,i}=p_{i}\Delta k\hat{x}+q_{i}\Delta k\hat{y}$, where *p_i_*, *q_i_* =  0, 1, ⋅⋅⋅, ensuring a uniform sampling in reciprocal space. Each channel maintains a constant wave vector magnitude $k_{0}=\frac{2\pi f_{0}}{c}$, where *c* is the speed of light in vacuum, guaranteeing consistent propagation characteristics across all channels. The schematic representation of this wave‐number decomposition is illustrated in Figure 3b. The focused field resulting from the application of PDID method to the optimized metasurface is presented in Figure 3c, which clearly demonstrates the effectiveness and reliability of the PDID method in accurately computing the near‐field distributions of large‐scale metasurfaces.

**Figure 4:**
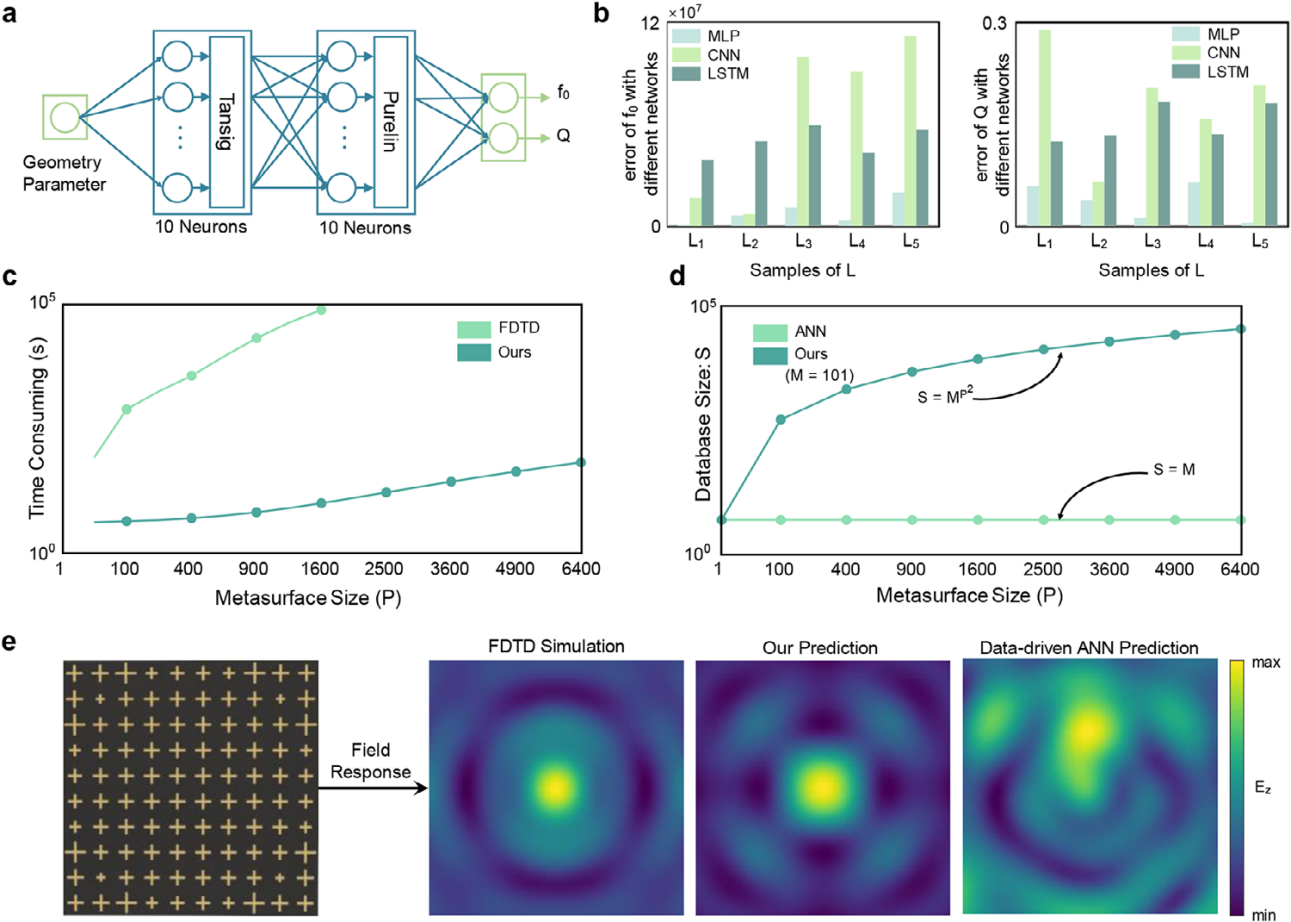
Performance comparison of the proposed PDID with traditional numerical and data‐driven ANN methods. a) Neural network used in PDID. b) Prediction error comparison among MLP, LSTM, and CNN for five different metasurface sizes, highlighting the superior accuracy of MLP. c) Computation time comparison between the FDTD numerical method and PDID, showing the significantly faster performance of the proposed PDID for large‐scale metasurfaces. d) Database size requirements for PDID and traditional data‐driven ANN methods, demonstrating that PDID can be used to reduce the reliance on extensive databases. e) The near‐field distributions of a light‐focusing metasurface evaluated by the accurate FDTD numerical method, PDID, and traditional data‐driven ANN method to validate the accuracy and reliability of the PDID method.

The design of the neural network under consideration is based on the data in the meta‐atom library. As shown in **Figure** **4**a, the hidden layer employs tansig as the activation function, while the output layer utilizes purelin. The Levenberg‐Marquardt algorithm is utilized to minimize the mean absolute error (MAE) during neural network training, ensuring efficient convergence and improved predictive accuracy (see Note S12, Supporting Information, for details). Its inputs are the geometry parameters of the meta‐atoms in the meta‐library; while its outputs are the resonance frequency *f*_0_ and the quality factor *Q*. We remark that 70% of data are randomly selected as the training set and the remaining 30% as the test set. Following a comparative analysis of the training performance across various neural network architectures (see Note S7, Supporting Information, for details), Multilayer Perceptron (MLP) is identified as the most suitable model for achieving the design objectives of this study. The comparative results shown in Figure 4b present bar charts of the loss values for two output parameters across three distinct network configurations at five test points. The results clearly indicate that the testing error associated with MLP is substantially lower than that observed in the other two network architectures. Consequently, MLP is selected as the optimal architecture for this design.

Compared with conventional EM numerical methods, PDID achieves a substantial reduction in the computational time while preserves the accuracy of predicting the near‐field characteristics of large‐scale metasurfaces. As shown in Figure 4c, the computational time for the finite‐difference time‐domain (FDTD) method rises sharply as the size of metasurface array increases, reaching an impractical threshold at an array size of 1600, namely 40×40 meta‐atoms. In contrast, the PDID method exhibits a much slower increase in computational time with the array size. Notably, at an array size of 6400, the PDID method can complete the predictions in 60 s. For large metasurface arrays comprising over 900 meta‐atoms, the PDID method achieves the prediction speeds that are several orders of magnitude faster than those of the conventional numerical methods. Undoubtedly, this highlights the advantages on designing speed of the PDID method over traditional numerical computation approaches. Additionally, the PDID method markedly reduces the complexity of database construction compared with the data‐driven artificial neural networks (ANNs). As shown in Figure 4d, the number of data samples required to build a comprehensive database using the PDID method is significantly lower than that required by data‐driven ANNs, especially as the metasurface size increases. The figure demonstrates that the number of data samples needed for the data‐driven ANNs to construct a complete database grows exponentially with the array size. In contrast, the dataset size for the PDID method only depends on the size of meta‐atom library rather the size of metasurface library, thereby eliminating the need to create new databases for different metasurface sizes. PDID significantly mitigates the data dependency of data‐driven ANNs, demonstrating strong generalization capabilities.

When predicting the EM responses of metasurfaces at new incident angles, the data‐driven ANNs require the construction of new databases, whereas the PDID method can accomplish this task without new databases and training. These advantages underscore the superior versatility of the PDID method, which is expected to facilitate its broad application across diverse metasurface designs, ensuring efficient design performance regardless of the array size. Figure 4e presents a comparison of the predicted results for a metasurface designed to achieve a focusing function using different computational methods. The PDID method yields results that are in good agreement with those obtained from the accurate and rigorous FDTD simulations. In contrast, the data‐driven ANN trained with the same number of samples as PDID fails to generate the desired focal point. This comparison further highlights the accuracy and reliability of the PDID method in predicting the near‐field distributions of metasurfaces.

### 2.4 Experimental Validations

**Figure 5:**
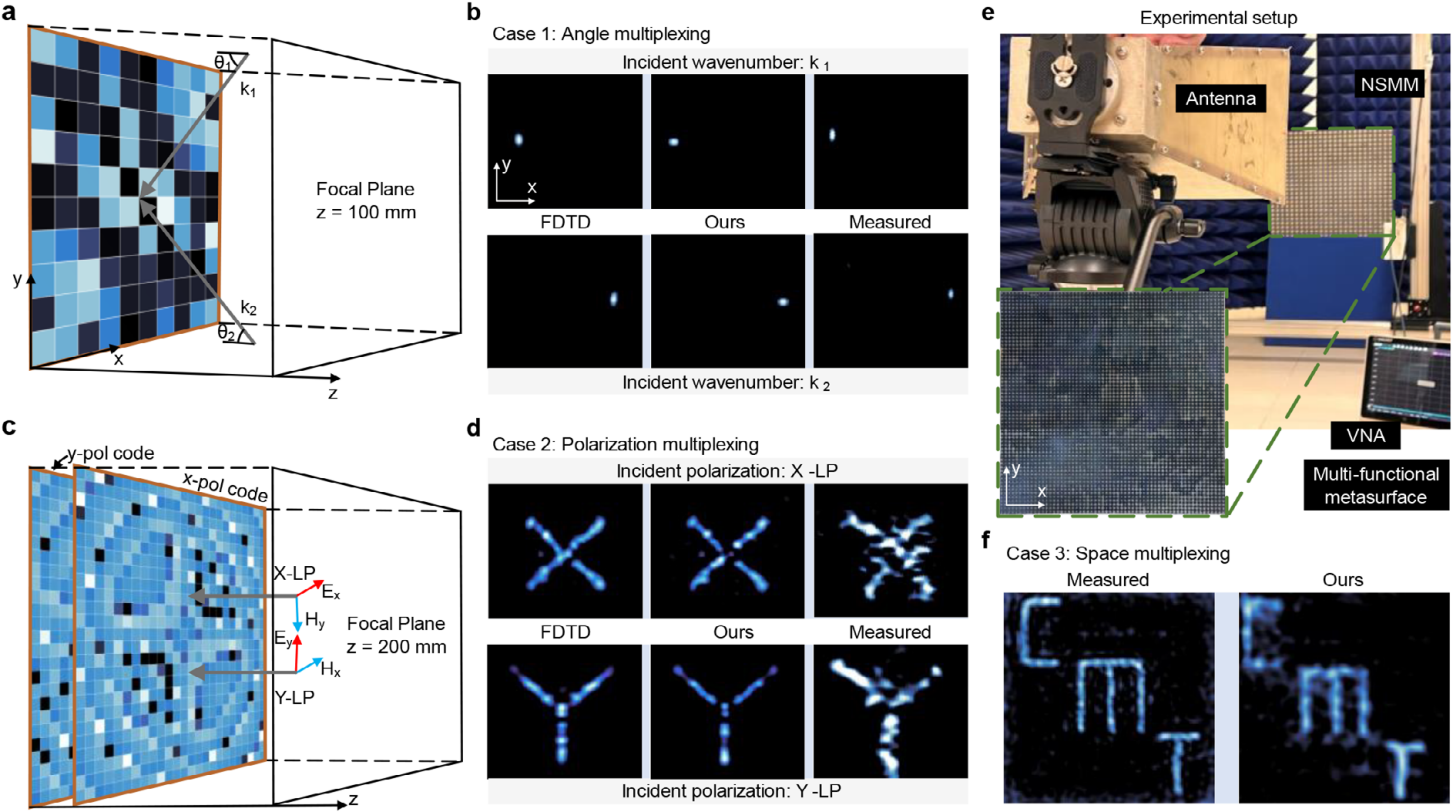
Experimental validation of MDOF multiplexed metasurfaces designed by PDID. a) Schematic of an angle‐multiplexed focusing metasurface. b) Near‐field comparison of the FDTD‐simulated, PDID‐predicted, and experimental results for angle‐multiplexed focusing under different incident angles. c) Schematic of a polarization‐multiplexed holographic metasurface. d) Near‐field comparison of the FDTD‐simulated, PDID‐predicted, and experimental results for polarization‐multiplexed holography under different polarization excitation. e) Photograph of the experimental setup for near‐field measurements (inset: a space‐multiplexed metasurface). f) Near‐field comparison of the PDID‐predicted and experimental results for space‐multiplexed holography, highlighting the accuracy and efficiency of the PDID method in large‐scale metasurface design.

To demonstrate the practical applicability of PDID in designing the MDOF multiplexed metasurfaces, we conducted a series of experiments using a scanning near‐field microwave microscope (SNMM) and a vector network analyzer (VNA). The experimental results of the MDOF multiplexed metasurfaces (refer to the Supporting Information for the detailed structures) are presented in **Figure** **5**. In the design, the optimization variables are defined as the arm lengths of each unit cell in an N×N array, denoted as $L=\{L^{\left(1\right)},L^{\left(2\right)},\text{…},L^{\left(N^{2}\right)}\}$. Starting from an initial solution, the variables are iteratively updated, with the discrepancy between the near‐field results obtained at each iteration and the target results serving as the objective function for optimization. For different scenarios, customized objective functions are formulated to achieve optimal optimization outcomes, ensuring efficiency and precision in the design process (see Note S10, Supporting Information, for details). This approach has been successfully implemented in the design of several metasurfaces, as detailed in the following experimental examples.

To initially assess the robustness of the PDID method, we designed a metasurface with 10×10 meta‐atoms to implement angle‐multiplexed focusing. The geometric size encoding diagram of the metasurface is shown in Figure 5a, the incident angle is 45° and − 45°. The experimental results, PDID predicted results, and numerical results obtained from FDTD simulations exhibit a high degree of consistency. This excellent agreement substantiates the reliability of the PDID method. Furthermore, the PDID method demonstrates remarkable adaptability to variations in incident angles, underscoring its robustness in handling diverse wavefront excitation conditions. To further evaluate the performance of the PDID method on larger‐scale metasurfaces, we developed a polarization‐multiplexed holographic metasurface. As shown in Figure 5c, the geometric parameters of two arms of the cross‐shaped metasurface are mutually independent, allowing for distinct encoding schemes when multiplexing in the X and Y polarizations. Under x‐polarized incidence, the metasurface generates an “X” hologram; while under y‐polarized incidence, generates a “Y” hologram. The results presented in Figure 5d demonstrate a high degree of consistency among experimental, PDID‐predicted, and simulated results, thereby substantiating the effectiveness of the PDID method. Notably, the meta‐atoms employed in this study feature unequal arm lengths, leading to excellent isolation under the x‐ and y‐polarized incident waves. Consequently, a database constructed from meta‐atoms with equal arm lengths remains robust enough to accurately predict the behaviour of such metasurfaces. This finding highlights the versatility and reliability of the proposed PDID method in complex metasurface designs.

More importantly, we designed and experimentally tested a holographic metasurface with 60×60 meta‐atoms, as shown in Figure 5e. For such a large‐scale metasurface, the FDTD simulations require several hours of computation, and the design optimization process can span several months, rendering it impractical for real‐world applications. In contrast, the PDID method computes the near‐field responses of this large‐scale metasurface in just 1 min, with the optimization process completed in two days. This represents a substantial advancement in practical metasurface engineering. Under the normal incidence, the metasurface generates a holographic “CMT” hologram, as shown in Figure 5g. These cases effectively demonstrate the versatility and computational efficiency of the PDID framework. A notable advantage of the PDID method over the conventional FDTD methods is its significant reduction (several orders of magnitude) in computational time, while maintaining high accuracy in predicting metasurface responses.

## 3 Conclusion

In this study, we introduced a novel physics‐driven intelligent method for the design of multi‐degree‐of‐freedom multiplexed metasurfaces, demonstrating its potential for accurate and flexible near‐field manipulation. By integrating the physical prior knowledge through the coupled mode theory into a neural network framework, the proposed PDID method significantly reduces the computational costs and enhances the design versatility compared to the traditional numerical methods and data‐driven intelligent designs. Our experimental validations, including angle‐multiplexed focusing, polarization‐multiplexed holography, and large‐scale space‐multiplexed metasurfaces, confirm the high accuracy and efficiency of the proposed PDID method. This interdisciplinary approach not only addresses the challenges of complex MDOF metasurface design but also offers a scalable solution for practical applications in the EM stealth, real‐time metasurface reconstruction, and advanced optical systems. Furthermore, the PDID framework can offer valuable insights for the design of multi‐mode resonant meta‐atoms. The current CMT algorithm adopted in PDID primarily focuses on the single mode behavior. Future work can be done in introducing an advanced CMT algorithm with multimode behavior and extending the PDID framework. The successful implementation of PDID highlights the potential of combining physical insights with machine learning, paving a way for future innovations in the computational science and materials engineering.

## 4 Experimental Section

### Meta‐Atom Design and Metasurface Fabrication

In this study, the proposed Multi‐Degree‐of‐Freedom (MDOF) metasurface employed a periodically arranged meta‐atom structure. The design aimed to achieve precise control over electromagnetic waves by finely tuning the geometric shape and arrangement of each meta‐atom. These meta‐atom characteristics were optimized through rigorous numerical simulations to ensure their performance under various electromagnetic conditions. The specific geometric parameters are presented in Figure 2, which includes key design elements such as the size, shape, spacing, and arrangement of the meta‐atoms. These parameters were determined by considering the radiation properties and mutual interactions of meta‐atoms during electromagnetic wave propagation.

The metallic structures of the metasurface were fabricated using conventional printed circuit board (PCB) technology, which provides high precision and reproducibility, ensuring the stability of large‐scale production. To further enhance the conductivity and corrosion resistance of the metal layers, the surface was treated with a gold‐plating process. The substrate material chosen was F4B, a low‐loss high‐performance dielectric material with a relative permittivity of 2.65 and an exceptionally low loss tangent of 0.001, which ensured minimal energy loss under high‐frequency conditions and thus improved the efficiency and stability of the metasurface. The thickness of the substrate and the design of the metallic patterns were critical. The top layer features finely designed metallic patterns, while the bottom layer was made of copper, ensuring excellent conductivity and mechanical stability. Both the top and bottom copper layers were 18 microns thick, which ensured stability and reliability during signal transmission.

For the ultra‐large metasurface designed in this study, the substrate thickness is *h*_1_ = 1.4 *mm*, the period of the metasurface unit is *p*_1_ = 8 *mm*, and the geometric parameters of the metal pattern are *w*_1_ = 0.6 *mm*. Other types of metasurfaces adopt different design parameters, with the substrate thickness set at *h*_2_ = 2 *mm*, the metasurface unit period at *p*_2_ = 20 *mm*, and the geometric parameters of the metal pattern at *w*_2_ = 2 *mm*. These different combinations of parameters not only adjust the electromagnetic response of the metasurface but also allow for the realization of various operating modes. The metasurface prototypes constructed in this study consist of 100, 400, and 3600 meta‐atom matrices, and the areas of the expanded metal backplates were 200×200 mm^2^, 500×500 mm^2^, and 480×480 mm^2^, respectively, to simulate the performance of metasurfaces at different scales.

### Simulations

All numerical simulations were performed using the commercial software CST Microwave Studio, ensuring the accuracy and reliability of the simulation results. To improve computational efficiency and simulation precision, high‐performance computational resources were employed, including an AMD Ryzen Threadripper 3960X24‐core processor (clocked at 3.8 GHz), two NVIDIA Tesla P40 GPUs, and one NVIDIA GeForce GT 710 GPU. This combination of resources enabled high‐precision electromagnetic simulations to be completed in a relatively short time. The simulations were conducted using the time‐domain solver in CST Studio Suite to analyze the electromagnetic characteristics of the meta‐atoms. In the simulations, open boundary conditions were applied along the x‐ and y‐axes, while open‐space boundary conditions were used along the z‐axis. This choice of boundary conditions minimizes the boundary effects, ensuring the most accurate representation of the electromagnetic characteristics. Additionally, the time‐domain solver was used to study the near‐field characteristics of the multi‐path metasurface, with open boundary conditions applied in all three dimensions (x, y, and z).

For frequency selection, the angle‐multiplexed metasurface and polarization‐multiplexed metasurface were both set to operate at a frequency of 7.30 GHz, as shown in Figure 5b,c. The large‐scale metasurface holography shown in Figure 4a operates at a frequency of 16 GHz. The focal length of the angle‐multiplexed metasurface was positioned 100 mm in front of the metasurface along the z‐direction. The imaging distances for the polarization‐multiplexed metasurface and the large‐scale metasurface holography were 200 and 250 mm, respectively. By adjusting these frequencies and focal lengths, the study could precisely verify the multiplexing characteristics and the effectiveness of the metasurface design.

### Experiments

To validate the accuracy and reliability of the simulation results, near‐field microwave scanning microscopy (NSMM) was used to measure the near‐field electromagnetic characteristics of the metasurfaces. The experimental system consists of an Agilent N5230C vector network analyzer and several phase‐stable coaxial cables. One coaxial cable was connected to the analyzer port as the excitation source and was linked to a broadband horn antenna (Model: XB‐CDL1‐18S, VSWR ≤ 2.5, SER: 16 092 802#) for signal transmission. The other coaxial cable connects to a coaxial probe (suitable for 1–18 GHz operation) mounted on a movable platform to detect the spatial electric field distribution on the metasurface.

During the experiment, the polarization direction and emission angle of the horn antenna were adjusted to measure the near‐field characteristics of the metasurface. The probe was mounted on a scanning platform, allowing for point‐by‐point measurements to obtain the spatial electric field pattern of a specific area. In the experiment, the distance from the probe tip to the sample surface was set at 100, 200, and 250 mm, ensuring comprehensive scanning at different heights. The scanned areas were consistent with those used in the numerical simulations, and the scan regions were 200 × 200, 500 × 500, and 480 × 480 mm^2^, with the minimum distance between adjacent points being 20 mm. These scan areas ensure that the experimental results match the simulation results with high accuracy. The frequency scanning range was selected as 6.8–7.8 and 15.5–16.5 GHz, with a total of 501 frequency points measured. These frequency points cover the key operating frequencies of the designed multiplexing metasurfaces, providing detailed data support for experimental comparison and the verification of the simulation results.

## Conflict of Interest

The authors declare no conflict of interest.

## Supporting information



Supporting Information

## Data Availability

The data that support the findings of this study are available from the corresponding author upon reasonable request.;
