# Supplementary material for: Multi‐Dimensional Multiplexed Metasurface for Multifunctional Near‐Field Modulation by Physics‐Driven Intelligent Design
Source: Adv Sci (Weinh). 2025 Apr 29;12(27):2503899. doi: 10.1002/advs.202503899 (PMC12279206; doi:10.1002/advs.202503899)
Supplement: Supplementary file 1 — Supporting Information [file ADVS-12-2503899-s001.docx]

**Supplementary Information for**

**Multi-Dimensional Multiplexed Metasurface for Multifunctional Near-Field Modulation by Physics-Driven Intelligent Design**

Jian Lin Su, Zi Xuan Cai, Yiqian Mao, Long Chen, Xin Yi Yu, Zhi Cai Yu, Qian Ma, Si Qi Huang, Jianan Zhang, Jian Wei You*, and Tie Jun Cui*

**This ﬁle includes:**

Supplementary Notes S1-S13

Supplementary Figures S1-S12

**Supplementary Note S1: Design of the proposed cross shaped meta-element**

To provide a comprehensive description of the cross shaped meta-element, we present a detailed analysis in Fig. S1. As illustrated in Fig. S1a, the meta-element is composed of three distinct layers. The first layer consists of a microwave plasmonic resonator made of copper film (see Fig. S1a), which is printed on the dielectric substrate made of F4B. The substrate has a relative permittivity of 2.65.

Our cross shaped meta-element is meticulously crafted using a multilayer Printed Circuit Board (PCB) lamination process. The specific PCB design is shown in Fig. S1. Figure S1b displays the three layers: top, bottom, and a dielectric layer. These layers are formed through precise processing techniques to ensure high quality performance. The top and bottom layers are made of metal and precisely designed to ensure the electromagnetic pathway.


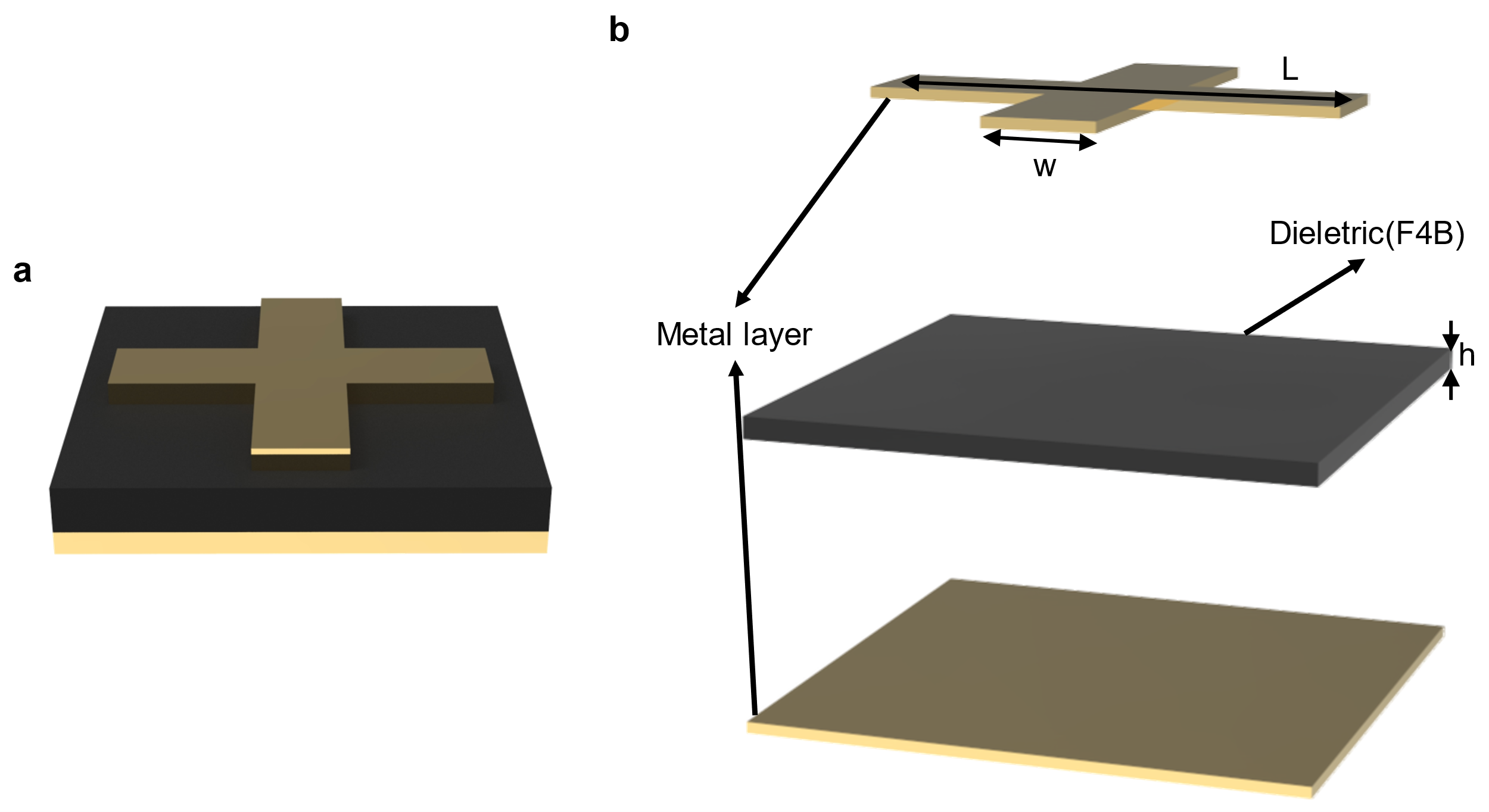


**Fig. S1 |** **Design schematic of the proposed cross shaped meta-element.** **a** Schematic of the structure of the cross shaped meta-element. **b** The meta-element comprises three layers, including a surface patch layer, dielectric substrate layer and the back patch layer, which surface metal patch layer and the back patch layer are consist of microwave plasma resonators made from copper film.

Additionally, we employed an Electroless Nickel Immersion Gold (ENIG) process, depositing a metallic compound on the PCB surface to form a metal coating layer. This coating not only serves as an anti-oxidation and anti-corrosion barrier but also significantly enhances solder joint strength, ensuring reliable electrical connections. The choice of the ENIG process further increases the overall stability and reliability of the circuit board, ensuring stable performance over long-term use.

**Supplementary Note S2** **: Design of the proposed cross shaped meta-element**

Under x- and y-polarized excitations, the cross-shaped metallic patch exhibits exceptional isolation between its two arms. As shown by S2, under x-polarized excitation, only the metallic arms aligned with the x-direction are activated, generating a surface electric field. Similarly, under y-polarized excitation, only the metallic arms aligned with the y-direction are activated to produce a surface electric field. This phenomenon remains consistent across metallic patches of varying sizes, significantly reducing the difficulty of designing large-scale polarization-multiplexed metasurface arrays in the following design procedure.


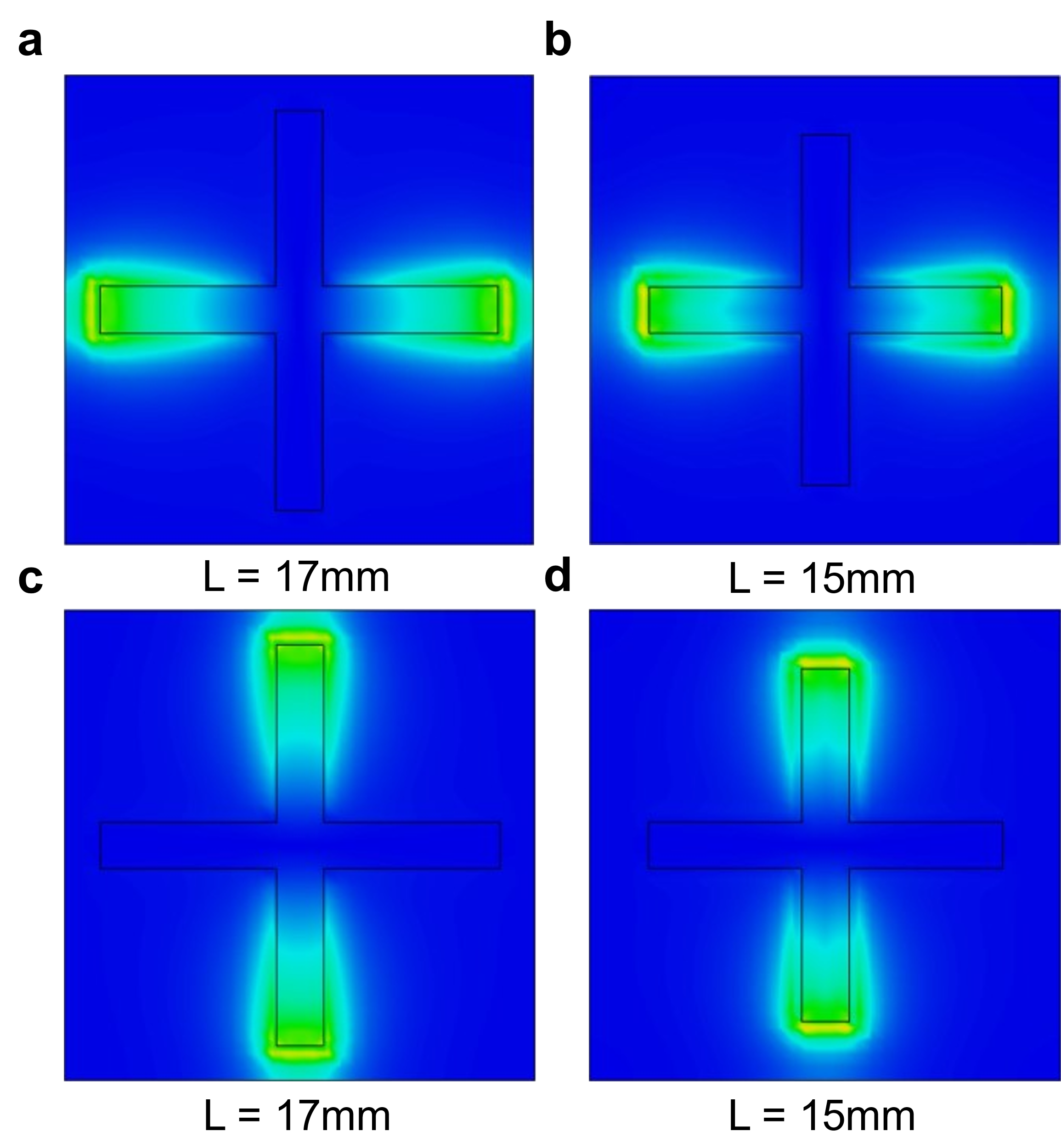


**Fig. S2 | Schematic diagram of the surface electric field distribution of the metallic patch under X and Y polarizations.** **a b** Surface electric field distribution of a metallic patch with arm lengths of $17mm$ and $15mm$ under X-polarized incident wave. **c d** Surface electric field distribution of a metallic patch with arm lengths of $17mm$ and $15mm$ under Y-polarized incident wave.

**Supplementary Note S3: Introduction to the Principles of Coupled Mode Theory (CMT)**

Here, the Coupled Mode Theory (CMT) employed in this work will be introduced. The coupled mode equations for periodic structures are given by

$$\begin{aligned} \left\{ \begin{aligned} \frac{d\boldsymbol{a}}{dt}&=\left( j\boldsymbol{\Omega}-\boldsymbol{\Gamma} \right)\boldsymbol{a}+\boldsymbol{D}^{T}\left| s_{+} \right\rangle\\ \left| s_{-} \right\rangle&=\boldsymbol{C} \left| s_{+} \right\rangle+\boldsymbol{Da} \end{aligned} \right.\#\left( 3-1 \right) \end{aligned}$$

the mode amplitudes can be expressed as

$$\boldsymbol{a}=\left( \begin{matrix} a_{1} \\ a_{2} \\ \vdots\\ a_{n} \end{matrix} \right)$$

the amplitude of the incident field can be expressed as

$$\left| s_{+} \right\rangle= \left( \begin{matrix} s_{1+} \\ s_{2+} \\ \vdots\\ s_{m+} \end{matrix} \right)$$

from channels 1 to m, with the coupling matrix

$$\boldsymbol{D}=\left[ \begin{matrix} k_{11} & k_{12} & \cdots& k_{1n} \\ k_{21} & k_{22} & \cdots& k_{2n} \\ \vdots& \vdots& \ddots& \vdots\\ k_{m1} & k_{m2} & \cdots& k_{mn} \end{matrix} \right]$$

the scattered wave coupled with the incident wave

$$\left| s_{-} \right\rangle=\left( \begin{matrix} s_{1-} \\ s_{2-} \\ \vdots\\ s_{m-} \end{matrix} \right)$$

Assuming the incident energy at the initial time $t=0$ is $\left| s_{+} \right\rangle=0$, at $t>0$, the mode amplitudes in the resonant system begin to decay, and are given by

$$\begin{aligned} \begin{aligned} \frac{d\left( \boldsymbol{a}^{\boldsymbol{+}}\boldsymbol{a} \right)}{dt}&=\frac{d\boldsymbol{a}^{\boldsymbol{+}}}{dt}\boldsymbol{a}+\boldsymbol{a}^{\boldsymbol{+}}\frac{d\boldsymbol{a}}{dt} \\ &=\boldsymbol{a}^{\boldsymbol{+}}\left( -j\boldsymbol{\Omega}-\boldsymbol{\Gamma} \right)\boldsymbol{a}+\boldsymbol{a}^{\boldsymbol{+}}\left( j\boldsymbol{\Omega}-\boldsymbol{\Gamma} \right)\boldsymbol{a} \\ &=-2\boldsymbol{a}^{\boldsymbol{+}}\boldsymbol{\Gamma}\boldsymbol{a} \end{aligned}\#\left( 3-2 \right) \end{aligned}$$

where we use the fact that both $\Omega$ and $\Gamma$ are Hermitian matrices. On the other hand, the overall system is energy conserving, the decaying of the energy of the modes are due entirely to the scattering waves. Hence

$$\begin{aligned} \frac{d\left( \boldsymbol{a}^{\boldsymbol{+}}\boldsymbol{a} \right)}{dt}=-\left\langle s_{-} | s_{-} \right\rangle=-\boldsymbol{a}^{\boldsymbol{+}}\boldsymbol{D}^{\boldsymbol{+}}\boldsymbol{Da}\#\left( 3-3 \right) \end{aligned}$$

by combining equations (2) and (3), the following can be obtained

$$\begin{aligned} \boldsymbol{D}^{\boldsymbol{+}}\boldsymbol{D}=2\boldsymbol{\Gamma}\#\left( 3-4 \right) \end{aligned}$$

Therefore, Eq. (1) can be transformed into

$$\begin{aligned} \left\{ \begin{aligned} \frac{d\boldsymbol{a}}{dt}&=\left( j\boldsymbol{\Omega}-\frac{\boldsymbol{D}^{\boldsymbol{+}}\boldsymbol{D}}{2} \right)\boldsymbol{a}+\boldsymbol{D}^{T}|s_{+}\rangle\\ \left| s_{-} \right\rangle&=\boldsymbol{C}\left| s_{+} \right\rangle+\boldsymbol{Da} \end{aligned} \right.\#\left( 3-5 \right) \end{aligned}$$

For a resonant system with $N$ resonators, the coupling matrix can be described as

$$\boldsymbol{D}^{T}=\left( \begin{matrix} \sqrt{\frac{2\omega_{1}}{Q_{1}cos\theta_{1}}}e^{j\boldsymbol{k}_{\boldsymbol{1}}\boldsymbol{r}_{\boldsymbol{1}}} & \cdots& \sqrt{\frac{2\omega_{1}}{Q_{1}cos\theta_{m}}}e^{j\boldsymbol{k}_{\boldsymbol{m}}\boldsymbol{r}_{\boldsymbol{1}}} \\ \vdots& \ddots& \vdots\\ \sqrt{\frac{2\omega_{n}}{Q_{n}cos\theta_{1}}}e^{j\boldsymbol{k}_{\boldsymbol{1}}\boldsymbol{r}_{\boldsymbol{n}}} & \cdots& \sqrt{\frac{2\omega_{n}}{Q_{n}cos\theta_{m}}}e^{j\boldsymbol{k}_{\boldsymbol{m}}\boldsymbol{r}_{\boldsymbol{n}}} \end{matrix} \right)$$

where $\boldsymbol{\theta=}\left\{ \theta_{1}, \theta_{2}, \cdots, \theta_{M} \right\}$ represents the azimuth angles associated with the channels numbered from $1$ to $M$ respectively and satisfies$\sum_{m=1}^{M} \frac{1}{\cos\theta_{m}}=1$. $\boldsymbol{C}$ contains the background reflection and transmission coefficients in the absence of the resonators and it satisfies $\boldsymbol{C}\boldsymbol{D}^{*}=-\boldsymbol{D}$ due to time-reversal symmetry. The following equation can be derived according to eqs (5):

$$\begin{aligned} \left| s_{-} \right\rangle=\left( \boldsymbol{C}+\boldsymbol{D}\left( j\omega_{0}\boldsymbol{I}_{N\times N}-j\boldsymbol{\Omega}+\frac{\boldsymbol{D}^{+}\boldsymbol{D}}{2} \right)^{-1}\boldsymbol{D}^{T} \right)\left| s_{+} \right\rangle\#\left( 3-6 \right) \end{aligned}$$

where $I_{N\times N}$is the $N\times N$ identity matrix. Eqs (6) describes the coupling between the incident wave and scattering wave. The intensity of the far-field scattering wave at location $\boldsymbol{r}^{'}=(x^{'},y^{'},z^{'})$ can be easily calculated as

$$\begin{aligned} F\left( \omega_{0},\boldsymbol{r}^{'} \right)=\left| \boldsymbol{W}\left( \boldsymbol{r}^{\boldsymbol{'}} \right) \right|s_{+}\rangle\left. \right|^{2}\#\left( 3-7 \right) \end{aligned}$$

where $\boldsymbol{W}\left( \boldsymbol{r}^{'} \right)=(e^{-j\boldsymbol{k}_{1}\boldsymbol{r}^{'}}\boldsymbol{,}e^{-j\boldsymbol{k}_{2}\boldsymbol{r}^{'}}, \cdots, e^{-j\boldsymbol{k}_{M}\boldsymbol{r}^{'}}\boldsymbol{)}$ represents the propagation phase of the plane waves in each channels.

**Supplementary Note S4: Experimental measurements of multi-dimensional multiscale metasurfaces**

During the procedure of evaluating the multi-dimensional multiscale metasurfaces, a high-precision near-field scanning microwave microscopy(NSMM) is used to measure their intricate near-field EM response. The experimental setup incorporates an advanced vector network analyzer (VNA: Agilent N5230C) alongside phase-stable coaxial cables, which ensure the accuracy and reliability of signal transmission. For instrument connections, two coaxial cables are connected to the VNA ports. One cable acts as the excitation source and is directly connected to the antenna, ensuring effective signal input. The other cables is linked to a coaxial probe mounted on a high-precisiion movable stage, which facilitates high-resolution spatial electric field distribution detection. By finely adjusting the position of the probe in the x, y and z directions, we accurately measure all vector components of the electric field $\left( E_{x}, E_{y},E_{z} \right)$. The probe is mounted on a precision scanning platform, enabling point-by-point measurements to obtain detailed spatial electric field patterns in specific regions. This high-precision measurement technique ensures a profound understanding of the EM response of the multi-dimensional multiplexed metasurfaces.

**
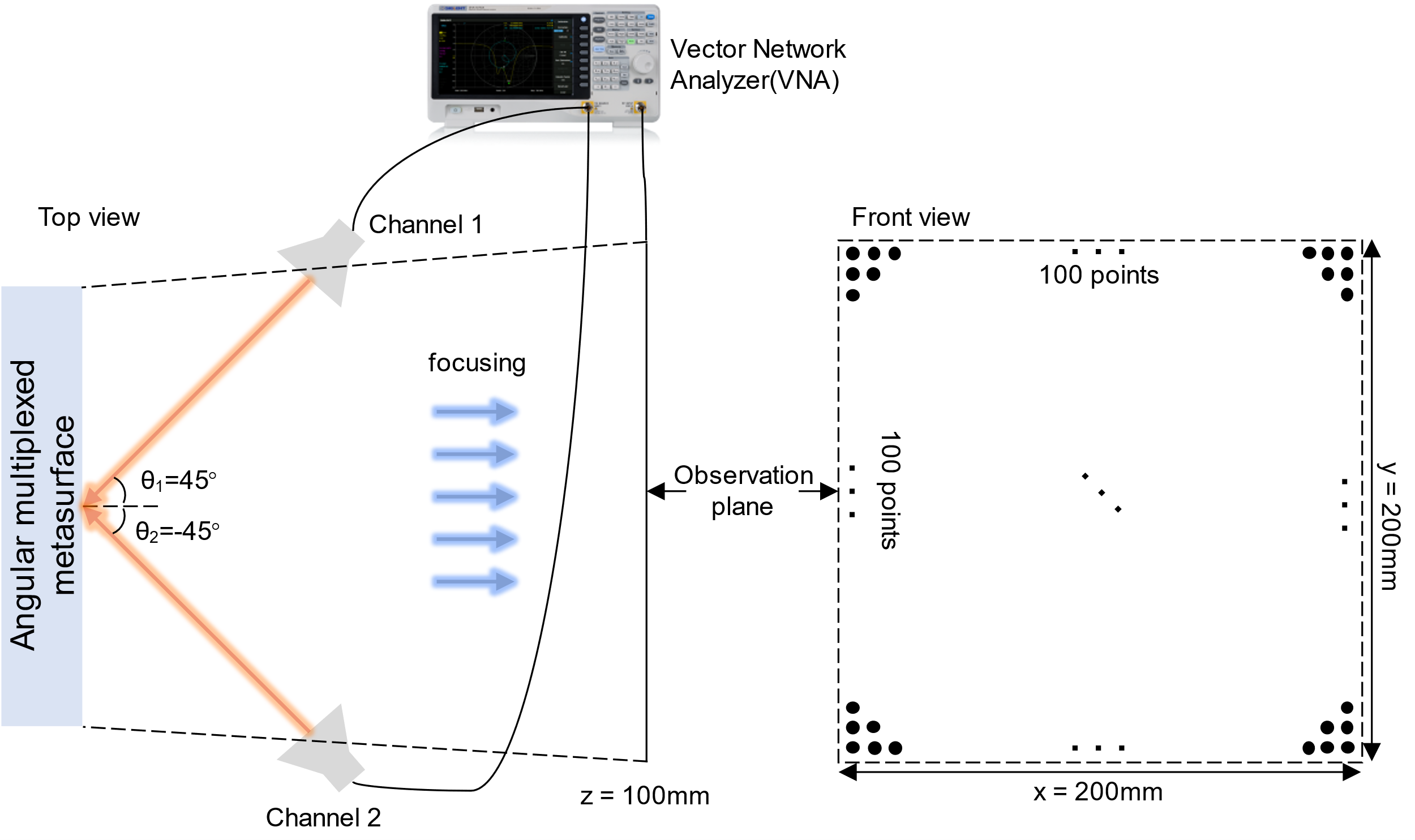
**

**Fig. S3 | Experimental setup for the measurement of the proposed angular multiplexed metasurface.** Prototype of the experimental setup, including the NSMM, a control computer, a VNA, a probe, an antenna and the metasurface. (left) Schematic of the 2D experimental configuration. The scanning area is a $200\times200 mm^{2}$ square positioned 100 mm in front of the samples surface. (right)

During the evaluation of the angular multiplexing properties of the metasurface, the position of the antenna is adjusted to enable incident angles of 45° and -45° with respect to the metasurface, as depicted in figure S3. The probe tip was maintained at a fixed distance of $2 mm$ from the sample surface to ensure consistent measurement conditions. A scanning plane of $200\times200 mm^{2}$ was employed to encompass a sufficient area, enabling a thorough assessment of the metasurface's performance under the specified incident angles. This setup allowed for precise characterization of the angular multiplexing behavior.

**
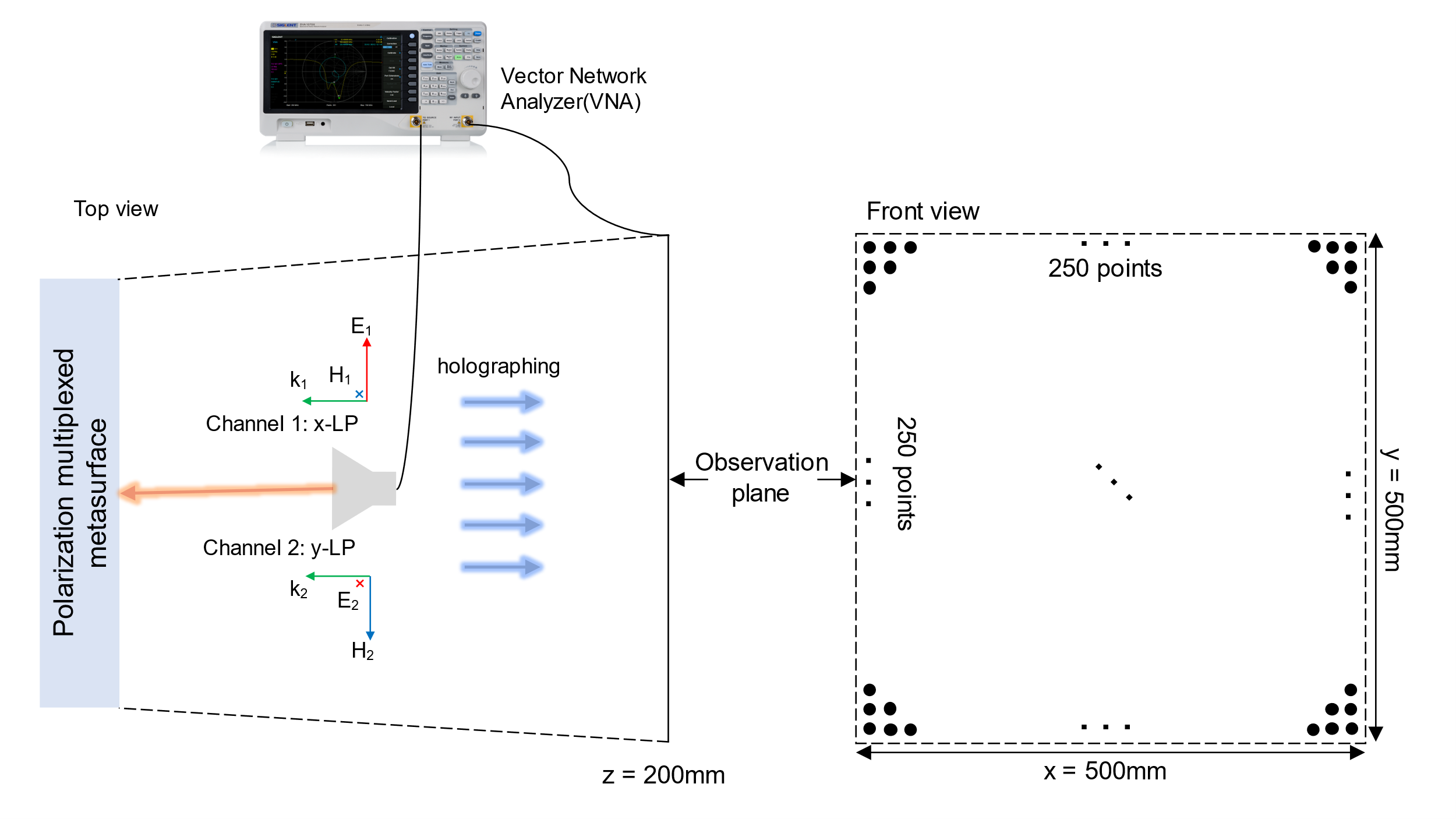
**

**Fig. S4 | Experimental setup for the measurement of the proposed polarization multiplexed metasurface.** Prototype of the experimental setup, including the NSMM, a control computer, a VNA, a probe, an antenna and the metasurface. (left) Schematic of the 2D experimental configuration. The scanning area is a $500\times500 mm^{2}$ square positioned $200 mm$ in front of the samples surface. (right)

In the assessment of the polarization multiplexed metasurface, the antenna was configured to switch between x-polarized and y-polarized incident waves, as shown in Figure S4. The probe tip was maintained at a constant distance of $2 mm$ from the surface of the sample to ensure measurement consistency. The scanning was conducted at a fixed plane located at $z=200 mm$, covering a $500\times500 mm^{2}$ area. This experimental setup enabled a comprehensive investigation into the metasurface's performance across different polarization states, providing detailed insights into its polarization multiplexing capabilities.

**
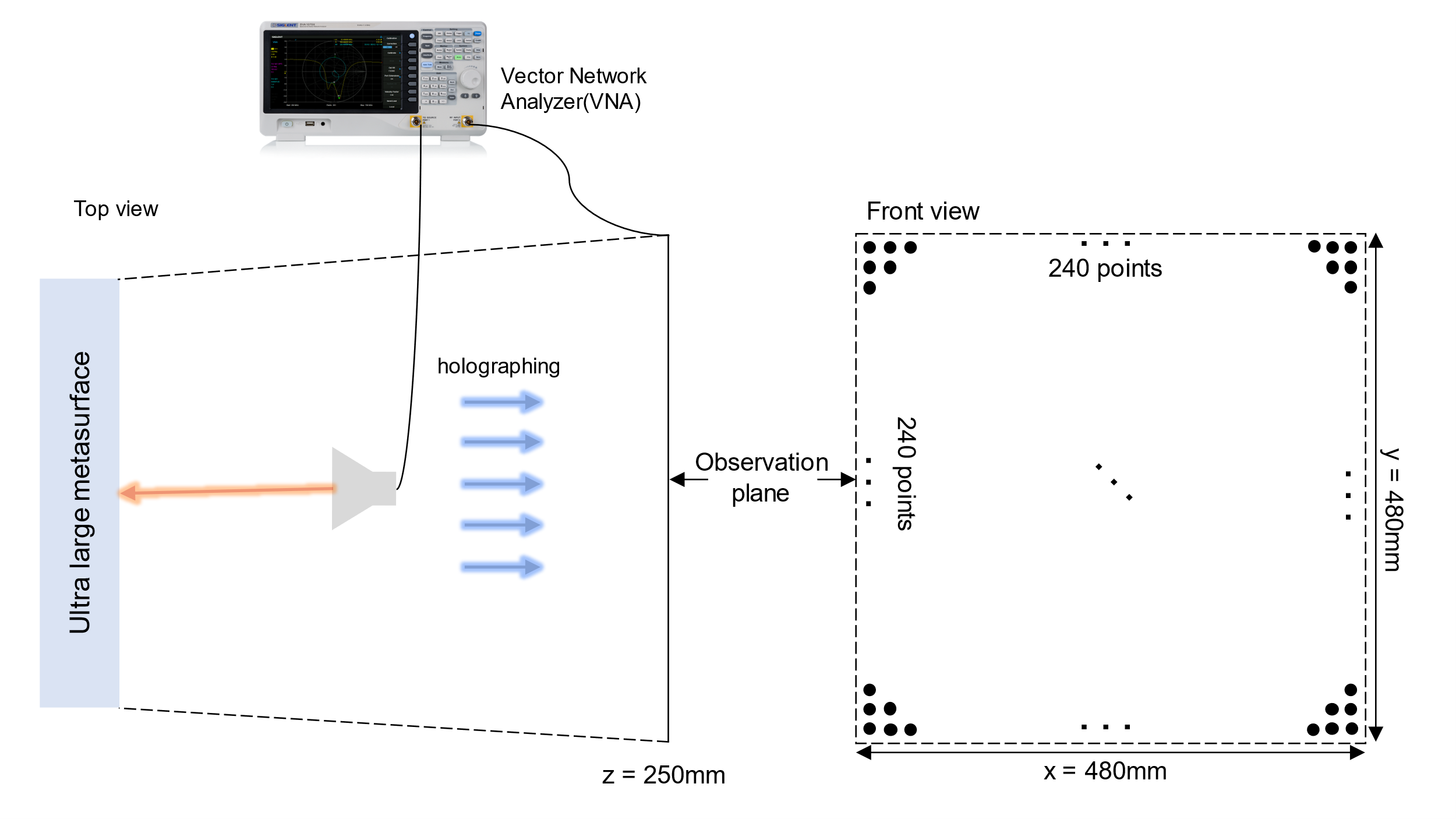
**

**Fig. S5 | Experimental setup for the measurement of the proposed ultra large metasurface.** Prototype of the experimental setup, including the NSMM, a control computer, a VNA, a probe, an antenna and the metasurface. (left) Schematic of the 2D experimental configuration. The scanning area is a $480\times480 mm^{2}$ square positioned $250 mm$ in front of the samples surface. (right)

For the measurement of ultra-large metasurfaces, the antenna was positioned to direct the incident waves onto the metasurface, as shown in Figure S5. The probe tip was maintained at a constant distance of $2 mm$ from the sample surface to ensure measurement consistency. Scanning was conducted at a fixed plane located at $z=250 mm$, with a scanning area of $480\times480 mm^{2}$. This setup enabled a comprehensive evaluation of the metasurface’s performance over a large spatial region, allowing for a detailed investigation of its characteristics.

**Supplementary Note S5: More detailed numerical simulations and calculation cases**

Figure S6 presents a series of additional computational and simulation cases designed using the Physics-Informed Intelligent Design (PDID) method, with a primary focus on its application for near-field shaping in the design of ultra-large metasurfaces. The cases featured in this figure demonstrate how PDID can be effectively used to create complex metasurface patterns that achieve precise control over electromagnetic fields in the near-field region. The patterns in the figure, inspired by traditional Mahjong symbols, serve as an illustrative example of how the PDID method can handle intricate geometries while meeting the functional requirements of near-field manipulation.

The PDID approach is a powerful tool for metasurface design, combining physical knowledge with intelligent algorithms to optimize the shape and functionality of metasurfaces. Unlike conventional design methods, PDID integrates physical principles governing electromagnetic field propagation, material properties, and boundary conditions directly into the optimization process. This physics-driven approach allows for the creation of metasurfaces that not only satisfy geometric constraints but also perform efficiently in terms of controlling electromagnetic wave behavior, particularly in the near-field region. Near-field shaping, which refers to the ability to manipulate the field distribution close to the surface of the metasurface, is a critical aspect of many optical and electromagnetic applications, such as beam focusing, field enhancement, and electromagnetic sensing.

One of the key advantages of PDID is its ability to design metasurfaces that influence near-field characteristics with high precision. The optimization process uses machine learning algorithms, informed by the physical laws governing the system, to explore a wide design space and identify configurations that produce the desired electromagnetic effects. This approach significantly reduces the time and computational resources typically required for such designs, while ensuring that the resulting metasurfaces meet both the functional performance and structural integrity required for practical implementation.

In the cases presented in Figure S6, the PDID method is applied to create metasurfaces that can shape and control the near-field electromagnetic distribution over large areas. This is particularly important in the design of ultra-large metasurfaces, where maintaining high design fidelity across extensive regions is a significant challenge. By incorporating physics-based constraints into the optimization, PDID ensures that complex patterns are not only accurately reproduced across large surfaces but also retain the required electromagnetic properties. The figure highlights how the method efficiently handles large-scale metasurface designs, making it suitable for applications in areas such as beam shaping, optical lenses, and near-field optical imaging, where precise control of the field distribution is essential.

Additionally, PDID’s flexibility in design is showcased in these expanded cases. The method can accommodate a variety of geometries, from simple to highly intricate, without compromising on performance. This makes PDID particularly well-suited for applications that demand large-scale metasurfaces with high precision, such as in the development of metasurfaces for antenna systems, imaging devices, or energy harvesting systems. In these cases, the PDID method optimizes the metasurface’s ability to control the electromagnetic field, achieving near-field enhancement or focusing in specific regions, which is critical for improving device performance.

In conclusion, Figure S6 emphasizes the substantial capabilities of the PDID method in the design of ultra-large metasurfaces with precise near-field shaping. By combining the power of physics-informed optimization with intelligent design algorithms, PDID allows for the creation of complex metasurfaces that manipulate electromagnetic fields with high precision and efficiency. The figure illustrates how PDID can be used to achieve both functional performance and geometric complexity, making it an ideal approach for the design of large-scale metasurfaces in a variety of scientific and engineering applications.


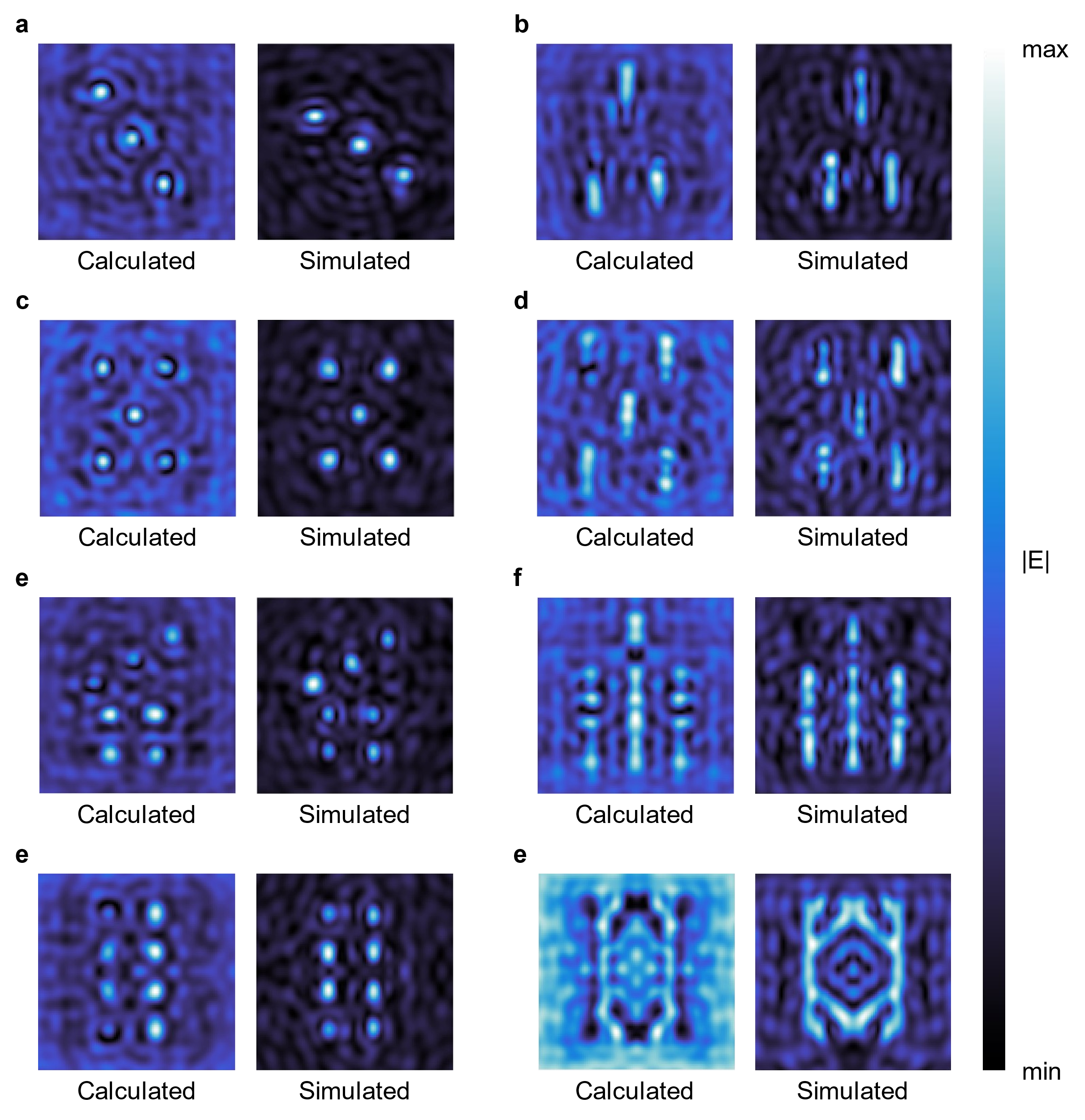


**Fig. S6 | Additional calculation and simulation result designed by PDID method.**

**Supplementary Note S6: EM responses in diverse geometry parameter**

In this supplementary note, we present a detailed analysis of the absorbance observed across various geometry parameters. Figure S7 plays a central role in this context, illustrating the spectral characteristic curves of the absorbance, which are crucial for understanding the EM behavior of different animal models. The figure highlights the magnitude of the absorbance, revealing significant variations across the different geometrical configurations. This data offers insight into the amplitude of the transmitted signal and how it is affected by changes in the model's geometry.

As shown in Figure S7, for varying geometry parameter $L$, both the resonant frequency of the unit cell and the magnitude of the resonant point in the absorbance undergo noticeable shifts. These disparities are not incidental; rather, they point to unique EM responses inherent to each specific geometry. Such variations in the resonant frequency and magnitude can be attributed to the distinct structural properties of each model, which influence the way electromagnetic waves interact with the system.

To ensure the accuracy and reliability of the simulation results, the absorbances for each geometry configuration are meticulously simulated, with each set consisting of 1001 frequency points spanning from 1 to 20 GHz. This high-resolution data collection approach is essential for capturing the full spectrum of electromagnetic responses and obtaining precise results. The use of the commercial EM simulation software, CST, enables us to extract these EM responses with high accuracy, offering valuable insights into the differences in electromagnetic behavior between the various animal models.

In summary, the variations in the absorbance across different geometrical configurations provide a comprehensive view of the EM characteristics and their dependence on the model's geometry. The high-resolution simulation and the use of advanced EM solvers like CST ensure that the findings are both reliable and significant, contributing to a deeper understanding of the electromagnetic responses of these models.


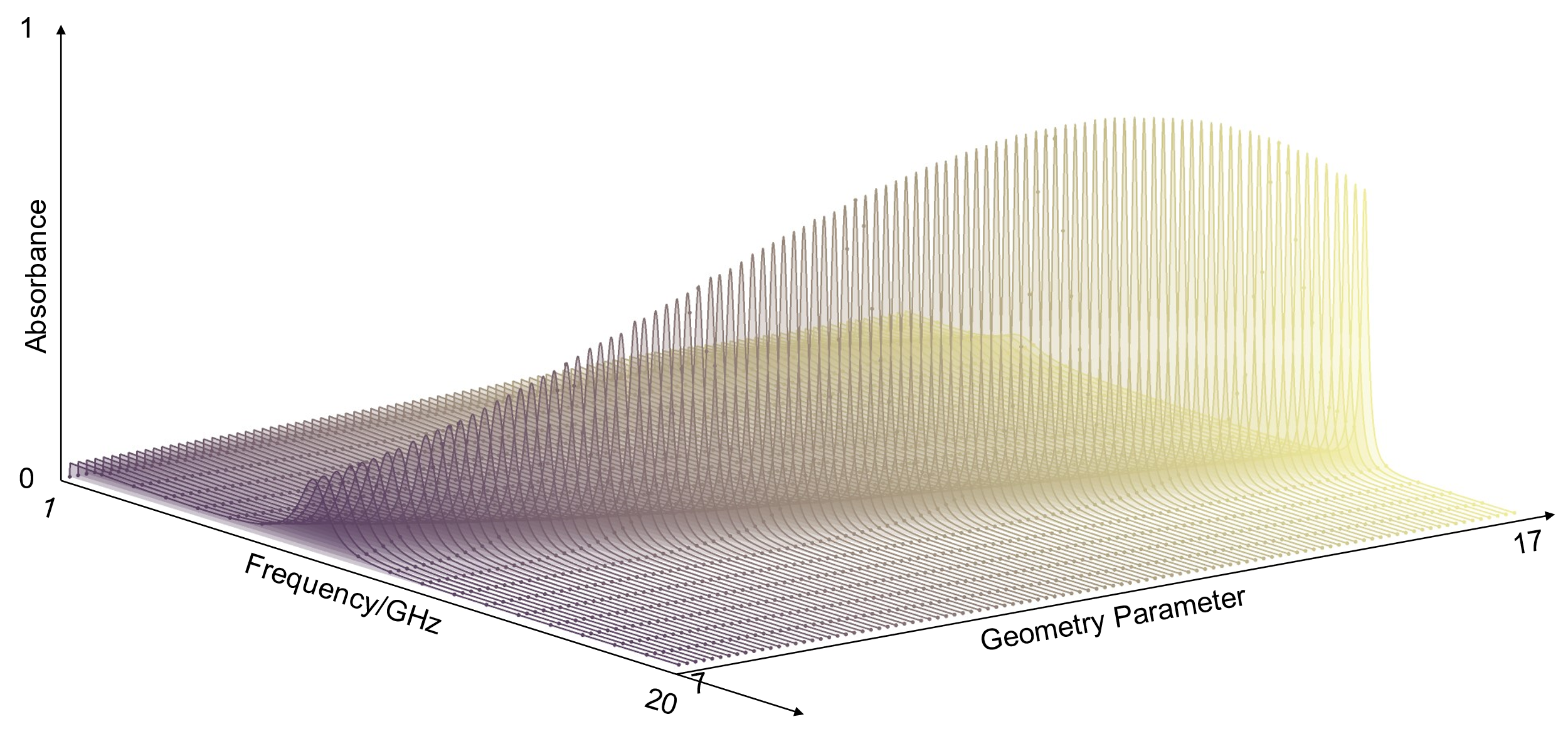


**Fig. S7 | Frequency domain data of EM responses from various geometry parameter.**

**Supplementary Note S7: Calculation method for resonant frequency** $\boldsymbol{f}_{\boldsymbol{0}}$ **and quality factor** $\boldsymbol{Q}$

**
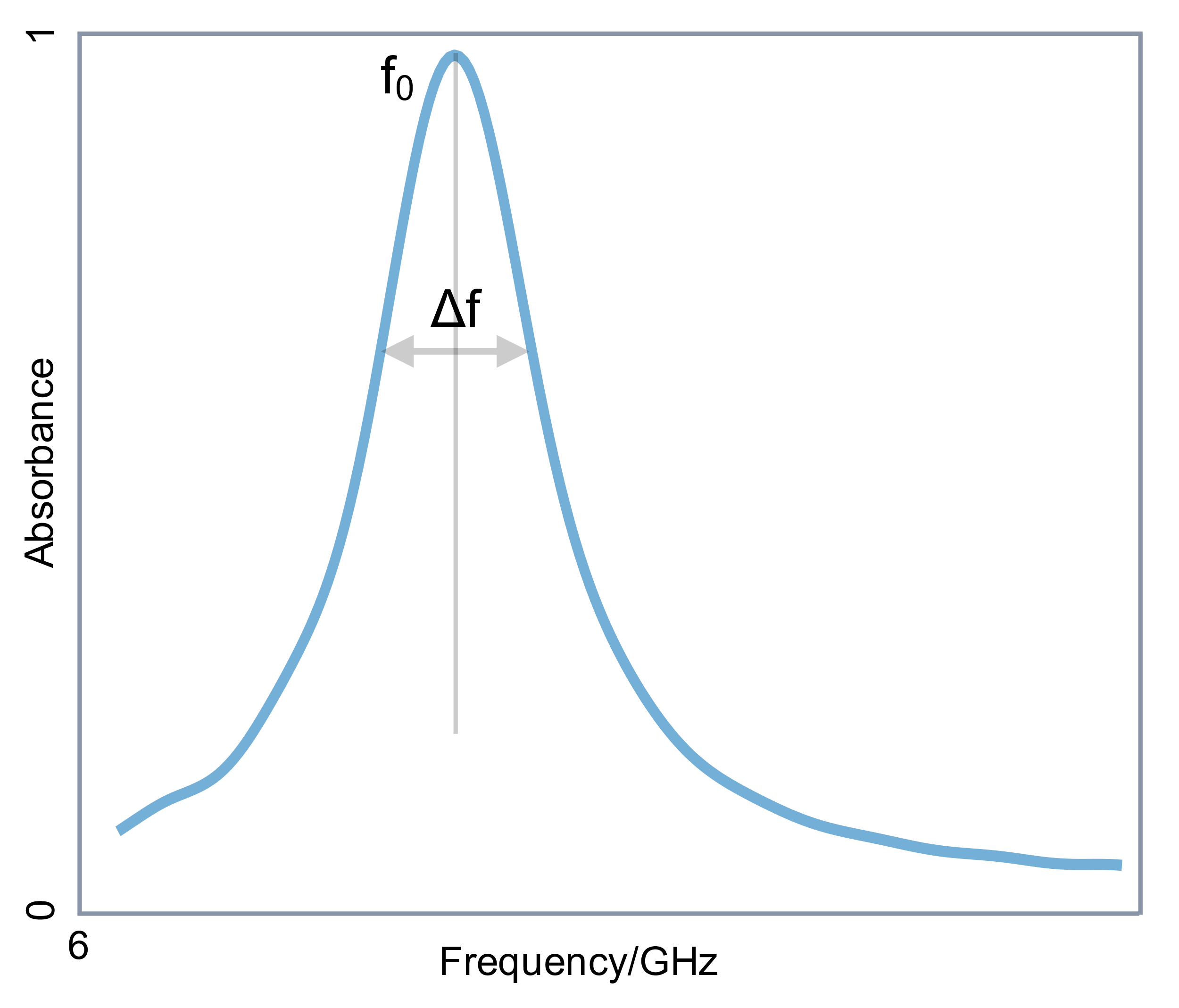
**

**Fig. S8 | Schematic diagram of the resonant frequency** $\boldsymbol{f}_{\boldsymbol{0}}$ **and** $\boldsymbol{\Delta}\boldsymbol{f}_{\boldsymbol{3}\boldsymbol{dB}}$**.**

The resonant frequency $f_{0}$ and quality factor $Q$ are fundamental parameters used to characterize resonant systems, such as metasurfaces. These parameters are crucial in understanding the system's response to EM excitation, providing key insights into the energy storage, resonance sharpness, and overall efficiency of the system.

The resonant frequency $f_{0}$ corresponds to the specific frequency at which a resonant structure, such as a metasurface or a resonator, stores the maximum amount of electromagnetic energy. It represents the point at which the system’s interaction with the incident EM wave is most efficient. In practical terms, this is often determined as the frequency where the system reaches its peak response, whether in terms of absorption, reflection, or transmission. In this study, the resonant frequency $f_{0}$ is obtained from the absorbance spectrum, which measures the energy absorbed by the system at different frequencies. The resonant frequency is mathematically defined as:

$$f_{0}=argmax(absorbance)$$

where the absorbance is the ratio of the absorbed energy to the total incident energy, and $f_{0}$ is the frequency at which the absorbance reaches its maximum value.

The quality factor $Q$ is a measure of the sharpness of the resonance and provides insight into the energy loss or dissipation within the resonant system. It quantifies the bandwidth of resonance relative to the resonant frequency, with a higher $Q$ indicating a narrower resonance and lower energy dissipation. A higher $Q$ implies that the system can store energy for a longer period, which is typically desired for applications such as sensing, filtering, and energy harvesting. The quality factor is defined as the ratio of the resonant frequency $f_{0}$ to the bandwidth $\Delta f$, where $\Delta f$ represents the full width at half maximum (FWHM) of the resonance curve, as shown in figure S8. Mathematically, it is expressed as:

$$Q=\frac{f_{0}}{\Delta f}$$

where $\Delta f$ is the frequency range over which the system’s response remains above half of its peak value, indicating the effective width of the resonance. A larger $Q$ value indicates a more selective resonance, and consequently, the system exhibits higher efficiency in terms of energy storage and lower energy loss during resonance.

Together, the resonant frequency $f_{0}$ and quality factor $Q$ are essential parameters for characterizing and optimizing resonant systems, particularly in applications involving metasurfaces and resonators where precise control over the EM field is crucial. These parameters help determine the operational frequency range and the effectiveness of the system in resonantly interacting with electromagnetic waves, influencing their design and performance in practical applications.

**Supplementary Note S8:** **The effect of different training epochs on the training results**


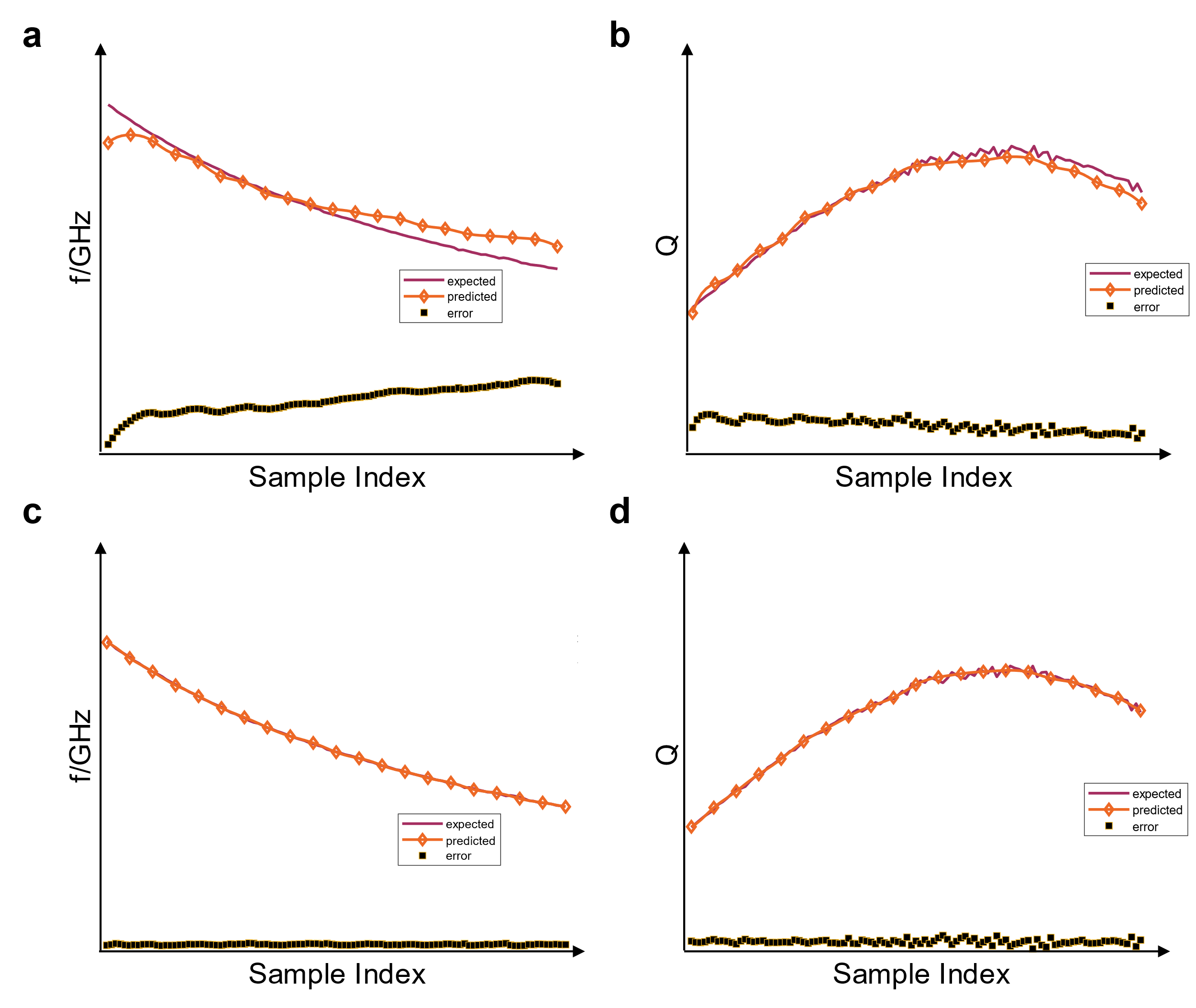


**Fig. S9 | The effect of different training epochs on the training results.** Comparison of the training results for resonant frequency (**a, c**) and quality factor (**b, d**) with the true results, for 10(**a, b**) and 100(**c, d**) training epochs, respectively.

In this section we explore the impact of the training epoch on the error of our designed MLP between the predicted data and the ground truth data. This parameter is of paramount importance during the training of deep learning models and significantly influence their performance.

Initially, a multilayer perceptron (MLP) model was trained for 10 epochs, completing the training process in just 5 minutes. However, upon evaluating the predicted results against the true values on the test set, it was evident that the performance of this network was suboptimal. The predictions showed considerable inaccuracies, with the error magnitude varying significantly across different samples. This variation in error suggests that the model was unable to effectively generalize to the test data, which made its performance unacceptable for practical use.

In response to these shortcomings, an alternative network was designed, which was trained for 100 epochs. The architecture of this network remained identical to the first, ensuring that the only difference was the number of training iterations. The training process for this model required approximately 1 hour to complete. After evaluating the predictions on the test set, it became clear that the network trained for 100 epochs exhibited much higher accuracy and consistency compared to the first network. The error across different samples was significantly reduced, and the model demonstrated much better generalization capabilities.

Despite the fact that the first network was trained in a relatively short time, the large prediction errors rendered it unsuitable for further use. The improved accuracy of the network trained for 100 epochs justifies the additional training time, as it resulted in a model with much better performance and reliability. Therefore, for the purpose of this study, the decision was made to use the network trained for 100 epochs, as its performance met the necessary criteria for both accuracy and consistency in predicting the desired outcomes.

This experience underscores the importance of balancing training time with model performance. While shorter training times may seem efficient, they can lead to subpar results, making it crucial to ensure that sufficient training epochs are used to optimize the model for accurate predictions.

**Supplementary Note S9: Introduction to the convolutional neural network (CNN) architecture for comparison**

**
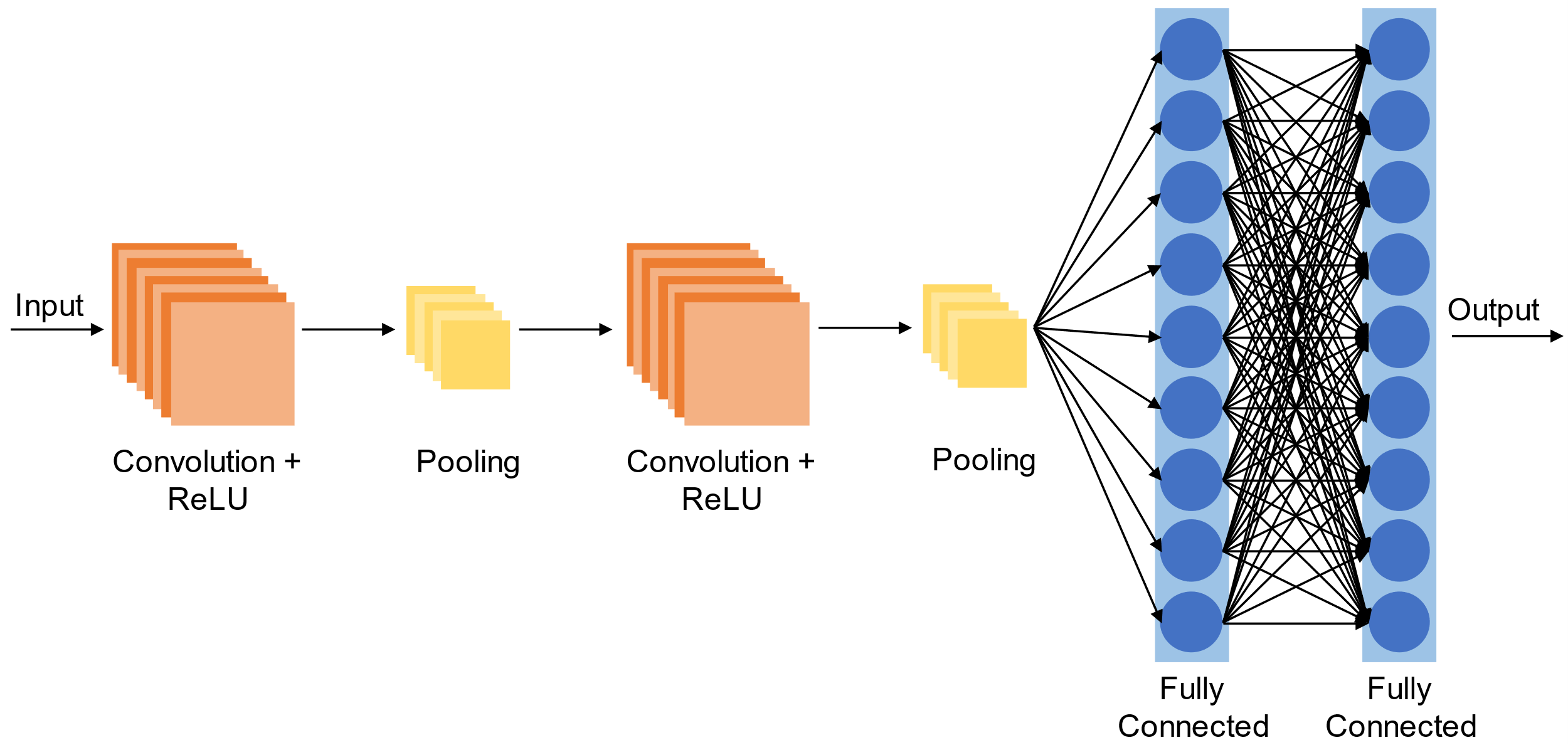
**

**Fig. S10 | The architecture of the CNN used in this work.** The CNN comprises two convolution layers, the activation function used is ReLU, and the network includes two fully connected layers, each consisting of 20 neurons.

Building upon the experimental data collected, we further assess the performance of a Convolutional Neural Network (CNN) in training and predicting the resonant frequency $f_{0}$ and quality factor $Q$ for various geometry parameters, extending the analysis beyond the MLP model shown in Fig. 4a. This investigation aims to explore how the CNN architecture can effectively model the relationship between the geometry of meta-units and their electromagnetic properties, focusing on improving prediction accuracy compared to the traditional MLP approach.

The input layer of the CNN architecture is designed to accept the geometry parameters of each meta-unit, which serve as the input features for the network. These geometry parameters are crucial as they directly influence the resonant behavior of the metasurfaces, and understanding this relationship is key to accurate prediction. The first convolutional layer in the network consists of 4 convolutional filters, each of size $3\times1$, with a stride of 1. This layer captures the initial spatial features of the input geometry, allowing the network to detect low-level patterns in the data that are important for further analysis.

The second convolutional layer follows, comprising 8 convolutional filters, also of size $3\times1$ and with a stride of 1. This layer is designed to capture more complex spatial relationships within the data, building upon the features extracted by the first layer. By stacking these convolutional layers, the network is able to progressively learn higher-level abstractions of the input data, enhancing its ability to model the underlying physical processes.

Following the convolutional layers, the network includes two fully connected layers, each containing 20 neurons. These fully connected layers are responsible for synthesizing the learned features and performing the final prediction. The dense layers ensure that the network can combine the extracted features from the convolutional layers in a meaningful way, enabling accurate regression or classification for the resonant frequency $f_{0}$ and quality factor $Q$.

**Supplementary Note S10:** **Objective function used in designing metasurfaces with PDID method**

The objective function plays a pivotal role in the optimization process, particularly in gradient-based algorithms. As a local optimization method, gradient descent efficiently converges to an optimal solution, making it well-suited for large-scale problems. However, due to its inherent tendency to settle in local optima, it may not always reach the global optimum. Notably, if the objective function exhibits a relatively flat landscape within its domain, the local optimum obtained is more likely to approximate the true global optimum, which is advantageous for optimization.

In the multi-angle focusing case, the primary objective is to maximize the field intensity at the focal point for each incident angle. This can be mathematically expressed as,

$$\begin{aligned} \mathcal{L}_{focus}=\sum_{\theta} \sum_{\left( x,y \right)\in focal point} \frac{1}{\left( E_{\theta} \right)^{2}} \#\left( 10-1 \right) \end{aligned}$$

To further refine the optimization, additional loss terms are introduced to suppress the field in non-target regions,

$$\begin{aligned} \mathcal{L}_{sidelobe}=\sum_{\theta} \sum_{\left( x,y \right)\notin focal point} \left( E_{\theta} \right)^{2}\#\left( 10-2 \right) \end{aligned}$$

Therefore, the total loss function for the multi-angle focusing scenario is formulated as,

$$\begin{aligned} \mathcal{L=}\alpha\mathcal{L}_{focus}+\beta\mathcal{L}_{sidelobe}\#\left( 10-3 \right) \end{aligned}$$

where $\alpha$ and $\beta$ are weighting factors that balance the contributions of each term. These parameters are carefully selected to achieve the desired trade-off between maximizing focal intensity, and reducing interference, thereby ensuring optimal performance of the designed system.

For the focusing case, we adopt the mean squared error (MSE) as the objective function to ensure that the optimized metasurface performs as expected under various polarization incidences. The specific formulation of the MSE used in the PDID method is given by,

$$\begin{aligned} \mathcal{L}_{MSE}=\frac{1}{N}\sum_{i=1}^{N} \left| E_{i}-E_{i, target} \right|^{2}\#\left( 10-4 \right) \end{aligned}$$

where $E_{i}$ is the *i*-th pixel point of the holographic field.

**Supplementary Note S11: Adjoint gradient descent method in PDID**

We start with the general mathematical expression of the gradient of the objective function$\mathcal{L}$ with respect to the parameters $\boldsymbol{L}$, which is given by

$$\begin{aligned} \frac{d\mathcal{L}}{d\boldsymbol{L}}=\frac{\partial\mathcal{L}}{\partial\boldsymbol{a}}\cdot\frac{d\boldsymbol{a}}{d\boldsymbol{L}}\#\left( 11-1 \right) \end{aligned}$$

From Supplementary Note S3, we can get

$$\begin{aligned} \left( j\omega_{0}\boldsymbol{I}-j\boldsymbol{\Omega+}\frac{\boldsymbol{D}^{\boldsymbol{+}}\boldsymbol{D}}{2} \right)\boldsymbol{a}=\boldsymbol{D}^{T}\left| s_{+} \right\rangle\#\left( 11-2 \right) \end{aligned}$$

Taking the derivative of the above equation with respect to $\boldsymbol{L}$, we obtain

$$\begin{aligned} \left( -j\frac{d\boldsymbol{\Omega}}{d\boldsymbol{L}}+\frac{1}{2}\cdot\frac{d\left( \boldsymbol{D}^{\boldsymbol{+}}\boldsymbol{D} \right)}{d\boldsymbol{L}} \right)\boldsymbol{a}+\left( j\omega_{0}\boldsymbol{I}-j\boldsymbol{\Omega+}\frac{\boldsymbol{D}^{\boldsymbol{+}}\boldsymbol{D}}{2} \right)\frac{d\boldsymbol{a}}{d\boldsymbol{L}}=\frac{d\boldsymbol{D}^{T}}{d\boldsymbol{L}}\left| s_{+} \right\rangle\#\left( 11-3 \right) \end{aligned}$$

After simplification, the objective function is expressed as

$$\begin{aligned} \frac{d\boldsymbol{a}}{d\boldsymbol{L}}\boldsymbol{=}\left( j\omega_{0}\boldsymbol{I}-j\boldsymbol{\Omega+}\frac{\boldsymbol{D}^{\boldsymbol{+}}\boldsymbol{D}}{2} \right)^{-1}\left( \frac{d\boldsymbol{D}^{T}}{d\boldsymbol{L}}\left| s_{+} \right\rangle\boldsymbol{+}j\frac{d\boldsymbol{\Omega}}{d\boldsymbol{L}}-\frac{1}{2}\cdot\frac{d\left( \boldsymbol{D}^{\boldsymbol{+}}\boldsymbol{D} \right)}{d\boldsymbol{L}} \right)\boldsymbol{\#}\left( 11-4 \right) \end{aligned}$$

Substituting the above result into Equation $(11-1)$, we derive

$$\begin{aligned} \frac{d\mathcal{L}}{d\boldsymbol{L}}=\frac{\partial\mathcal{L}}{\partial\boldsymbol{a}}\left( j\omega_{0}\boldsymbol{I}-j\boldsymbol{\Omega+}\frac{\boldsymbol{D}^{\boldsymbol{+}}\boldsymbol{D}}{2} \right)^{-1}\left( \frac{d\boldsymbol{D}^{T}}{d\boldsymbol{L}}\left| s_{+} \right\rangle\boldsymbol{+}j\frac{d\boldsymbol{\Omega}}{d\boldsymbol{L}}-\frac{1}{2}\cdot\frac{d\left( \boldsymbol{D}^{\boldsymbol{+}}\boldsymbol{D} \right)}{d\boldsymbol{L}} \right)\#\left( 11-5 \right) \end{aligned}$$

To avoid the computational burden of inverting large-scale matrices, the adjoint matrix $\boldsymbol{a}_{adj}$ is introduced

$$\begin{aligned} \boldsymbol{a}_{adj}=\frac{\partial\mathcal{L}}{\partial\boldsymbol{a}}\left( j\omega_{0}\boldsymbol{I}-j\boldsymbol{\Omega+}\frac{\boldsymbol{D}^{\boldsymbol{+}}\boldsymbol{D}}{2} \right)^{-1}\#\left( 11-6 \right) \end{aligned}$$

The adjoint equation can thus be formulated as

$$\begin{aligned} \left( j\omega_{0}\boldsymbol{I}-j\boldsymbol{\Omega+}\frac{\boldsymbol{D}^{\boldsymbol{+}}\boldsymbol{D}}{2} \right)\boldsymbol{a}_{adj}=\frac{\partial\mathcal{L}}{\partial\boldsymbol{a}}\#\left( 11-7 \right) \end{aligned}$$

By solving this adjoint equation iteratively to obtain $\boldsymbol{a}_{adj}$ and substituting it into Equation $(11-5)$

$$\begin{aligned} \frac{d\mathcal{L}}{d\boldsymbol{L}}=\boldsymbol{a}_{adj}\left( \frac{d\boldsymbol{D}^{T}}{d\boldsymbol{L}}\left| s_{+} \right\rangle\boldsymbol{+}j\frac{d\boldsymbol{\Omega}}{d\boldsymbol{L}}-\frac{1}{2}\cdot\frac{d\left( \boldsymbol{D}^{\boldsymbol{+}}\boldsymbol{D} \right)}{d\boldsymbol{L}} \right)\#\left( 11-8 \right) \end{aligned}$$

Then, by applying the chain rule of differentiation, we can compute the gradients efficiently

$$\begin{aligned} \frac{d\boldsymbol{D}}{d\boldsymbol{L}}=\frac{\partial\boldsymbol{D}}{\partial\omega}\frac{\partial\omega}{\partial\boldsymbol{L}}+\frac{\partial\boldsymbol{D}}{\partial Q}\frac{\partial Q}{\partial\boldsymbol{L}}\#\left( 11-9 \right) \end{aligned}$$

$$\begin{aligned} \frac{d\boldsymbol{\Omega}}{d\boldsymbol{L}}=\frac{\partial\boldsymbol{\Omega}}{\partial\omega}\frac{\partial\omega}{\partial\boldsymbol{L}}+\frac{\partial\boldsymbol{\Omega}}{\partial Q}\frac{\partial Q}{\partial\boldsymbol{L}}\#\left( 11-10 \right) \end{aligned}$$

the derivatives of $\omega$ and $Q$ with respect to $\boldsymbol{L}$ can be computed through the backpropagation process within the neural network, allowing for efficient gradient updates and optimization of the model parameters.

**Supplementary Note S12: Details on neural network**

In this paper, we utilized a multilayer perceptron to implement PDID. This network consists of a two-layer structure: the first layer is a hidden layer with 10 neurons, using the tansig activation function, while the second layer is the output layer, also comprising 10 neurons, with purelin as the activation function. The initial learning rate of the network is set to 0.25, and the training dataset consists of 101 samples.


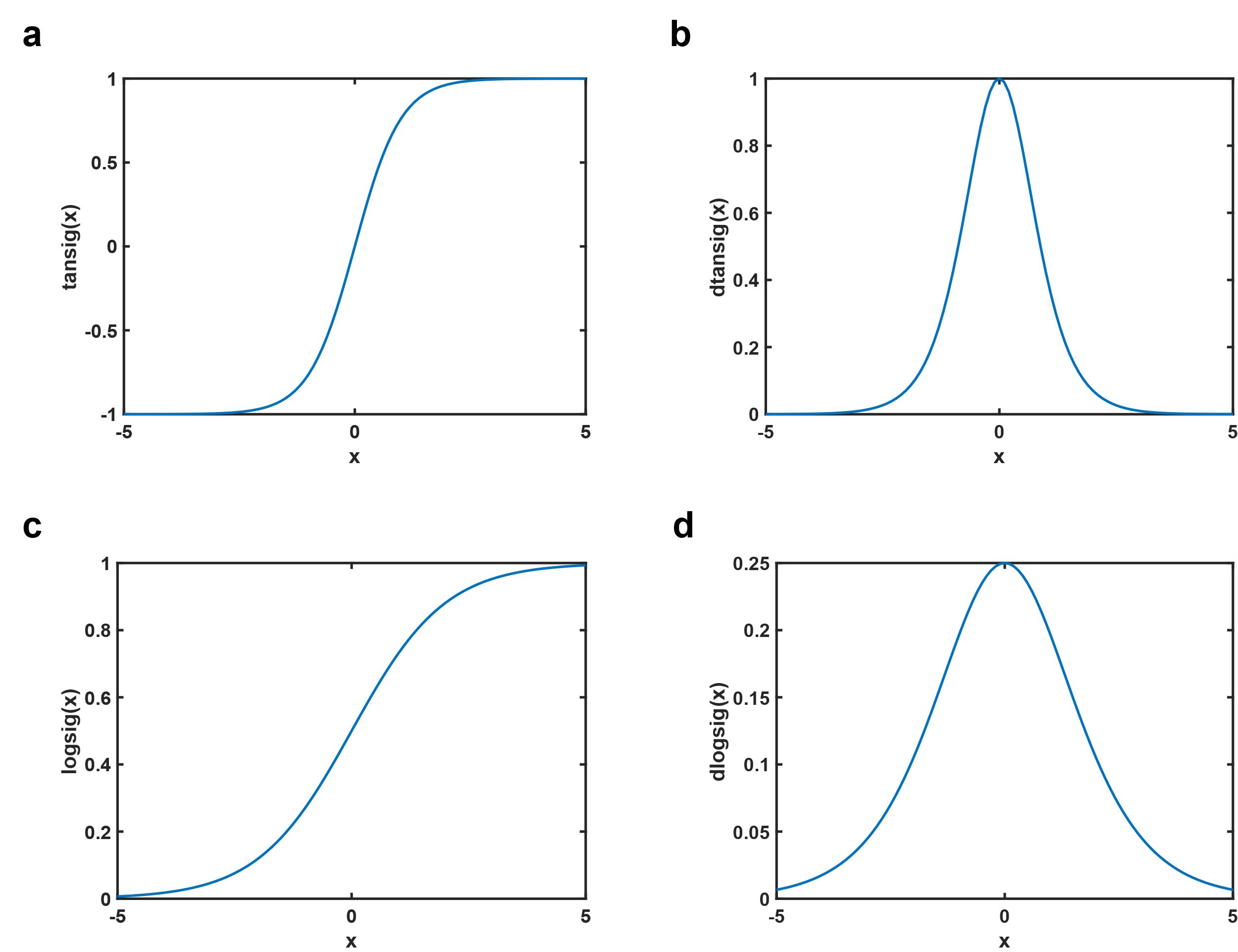


**Figure S11.** The activation functions tansig and logsig along with their derivatives. ((a) The function curve of tansig. (b) The derivative curve of tansig. (c) The function curve of logsig. (d) The derivative curve of logsig.

The tansig and logsig functions are among the most commonly employed activation functions in the hidden layers of backpropagation (BP) neural networks. While both are widely utilized due to their smooth, differentiable properties, they exhibit several key differences that influence their effectiveness in practical applications. The tansig and logsig functions are defined as follows:

$$\begin{aligned} f_{tansig}\left( x \right)=\frac{2}{1+e^{-2x}}-1 \#\left( 12-1 \right) \end{aligned}$$

$$\begin{aligned} f_{logsig}\left( x \right)=\frac{1}{1+e^{-x}} \#\left( 12-2 \right) \end{aligned}$$

As illustrated in Figure S11(a) and (c), the primary distinction between these two functions lies in their output ranges: the tansig function produces values within the symmetric interval [-1, 1], whereas the logsig function is confined to the asymmetric range [0, 1], This inherent asymmetry in the logsig function can introduce challenges in deep network training. Specifically, the nonzero mean of logsig outputs can lead to a phenomenon known as mean shift, which may systematically alter the distribution of activations across layers. This shift has the potential to affect the direction of weight updates during gradient descent, thereby impacting network convergence and stability. By contrast, the tansig function yields outputs centered more closely around zero, effectively reducing the extent of mean shift and enhancing numerical stability. Consequently, when employing tansig, the network is less susceptible to biased weight updates, ultimately contributing to a more balanced and efficient training process. The respective first-order derivatives of tansig and logsig functions are given by:

$$\begin{aligned} f_{tansig}^{'}\left( x \right)=1-f_{tansig}^{2}\left( x \right) \#\left( 12-3 \right) \end{aligned}$$

$$\begin{aligned} f_{logsig}^{'}\left( x \right)=f_{logsig}\left( x \right)\left( 1-f_{logsig}\left( x \right) \right)\#\left( 12-4 \right) \end{aligned}$$

As depicted in Figure S11(b) and (d), the derivative of the tansig function exhibits a broader range across its input interval [-1, 1] when compared to that of the logsig function. This characteristic implies that, during the training process, the tansig function allows for more substantial variations in gradient magnitudes across different input values. The increased range of derivatives enables more dynamic weight updates, which can accelerate convergence and enhance the overall efficiency of the learning process. In contrast, the relatively limited range of the logsig derivative may constrain gradient updates, potentially leading to slower convergence, especially in deep networks where small gradients can hinder effective weight adjustments.

Furthermore, in the neural network architecture proposed in this study, we employ the purelin function as the activation function in the output layer. The selection of purelin is motivated by its linear nature, which avoids the restrictive output range imposed by nonlinear activation functions. This choice is particularly beneficial when dealing with regression tasks or problems requiring unrestricted output values. Additionally, from an optimization perspective, using purelin in the output layer ensures stable gradient propagation during error backpropagation. The absence of saturation effects, which are commonly encountered with nonlinear activation functions, prevents gradient vanishing issues and facilitates more efficient learning. By maintaining a direct proportionality between the network's output and the weighted sum of activations from the previous layer, purelin enhances training efficiency while avoiding unnecessary increases in computational complexity.

Furthermore, the Levenberg-Marquardt (LM) algorithm is integrated into the PDID framework to minimize the mean absolute error (MAE) and enhance network training efficiency, as formulated below

$$\begin{aligned} argmin\frac{1}{n}\sum_{i=1}^{n} \left| y-f_{NN}\left( x \right) \right|\#\left( 12-5 \right) \end{aligned}$$

where $f_{NN}$ represents the neural network, while $x$ and $y$ denote its input and output parameters, respectively. The LM algorithm dynamically balances between gradient descent and the Gauss-Newton method, enabling adaptive optimization. This approach offers notable advantages, including rapid convergence and strong numerical stability, making it particularly well-suited for training small-scale neural networks.

**Supplementary Note S13: The influence of angle parameter selection on algorithm adaptability**

In this supplementary note, we systematically investigate the impact of oblique incidence on the adaptability of the PDID algorithm through extensive numerical calculations and full-wave simulations across a broad angular range. As shown in Figure S12, the PDID algorithm accurately reconstructs the electromagnetic field distributions obtained from CST simulations, demonstrating strong reliability and consistency across varying incidence angles. However, at large oblique angles, while unit-level predictions remain highly accurate, a noticeable degradation in system-level holographic imaging quality is observed. This phenomenon, evident in both numerical and simulation results, arises not from algorithmic limitations but from intrinsic constraints of the metasurface unit structure. As the incidence angle increases, higher-order diffraction effects and angular-dependent scattering become more pronounced, reducing the efficiency of wavefront manipulation and leading to deviations in the desired field distribution. By elucidating these fundamental limitations, this analysis provides valuable insights into the angular performance of metasurfaces and offers guidance for optimizing their design under oblique illumination conditions.


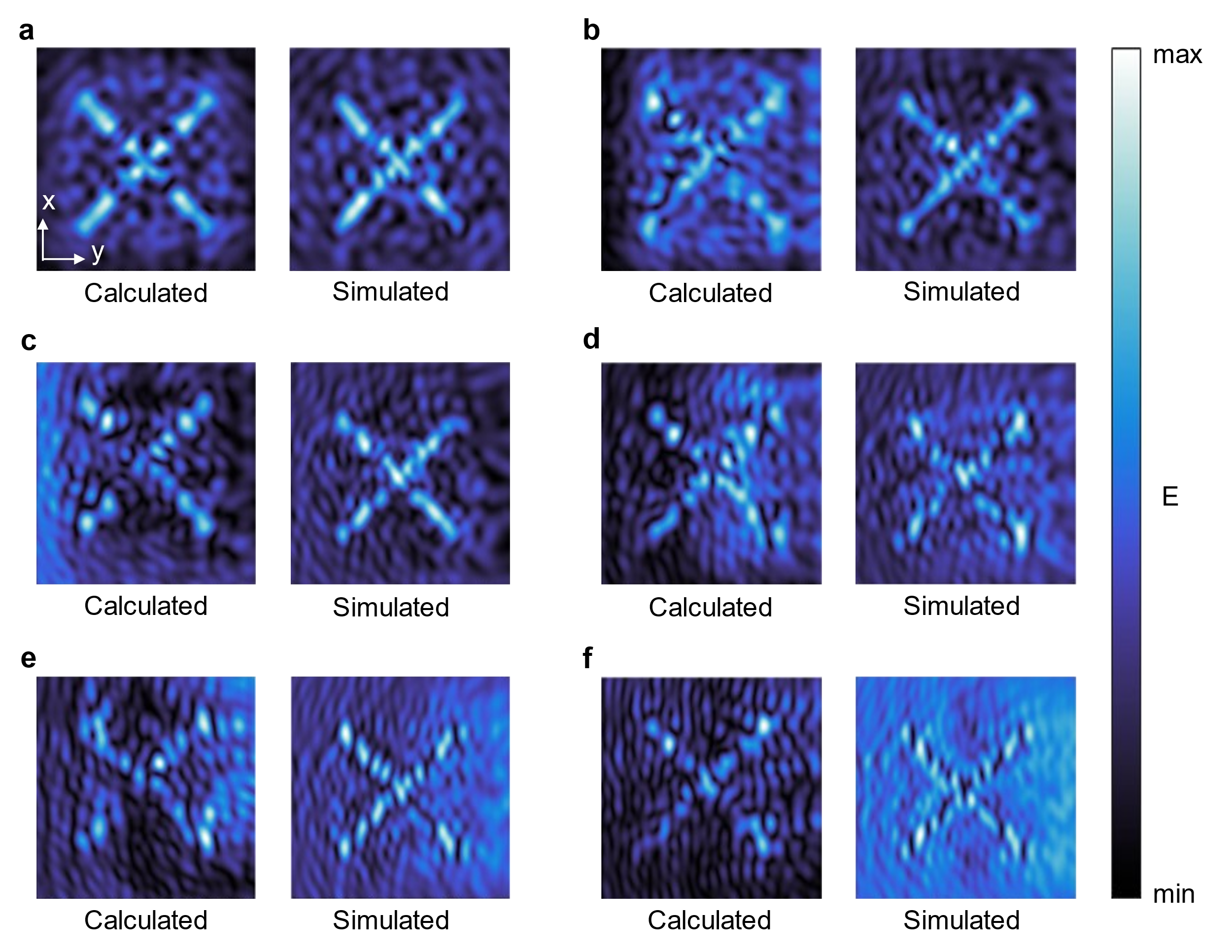


**Figure S12.** Calculation and simulation result designed by PDID method at different angles of oblique incidence, resonant frequency 7.3GHz, focal plane 200mm. (a) θ=0° (b) θ=15° (c) θ=30° (d) θ=45° (e) θ=60° (f) θ=75°
